# The dimeric deubiquitinase USP28 integrates 53BP1 and MYC functions to limit DNA damage

**DOI:** 10.1093/nar/gkae004

**Published:** 2024-01-16

**Authors:** Chao Jin, Elias Einig, Wenshan Xu, Ravi Babu Kollampally, Andreas Schlosser, Michael Flentje, Nikita Popov

**Affiliations:** Department of Medical Oncology and Pulmonology, University Hospital Tübingen, Otfried-Müller-Str 14, 72076 Tübingen, Germany; DFG Cluster of Excellence 2180 ‘Image-guided and Functionally Instructed Tumor Therapies’ (iFIT), University of Tübingen, Tübingen, Germany; Department of Medical Oncology and Pulmonology, University Hospital Tübingen, Otfried-Müller-Str 14, 72076 Tübingen, Germany; DFG Cluster of Excellence 2180 ‘Image-guided and Functionally Instructed Tumor Therapies’ (iFIT), University of Tübingen, Tübingen, Germany; Department of Radiation Oncology, University Hospital Würzburg, Josef-Schneider-Str. 2, 97080 Würzburg, Germany; Department of Medical Oncology and Pulmonology, University Hospital Tübingen, Otfried-Müller-Str 14, 72076 Tübingen, Germany; DFG Cluster of Excellence 2180 ‘Image-guided and Functionally Instructed Tumor Therapies’ (iFIT), University of Tübingen, Tübingen, Germany; Rudolf Virchow Center, Center for Integrative and Translational Bioimaging, University of Würzburg, Josef-Schneider-Str 2, 97080 Würzburg, Germany; Department of Radiation Oncology, University Hospital Würzburg, Josef-Schneider-Str. 2, 97080 Würzburg, Germany; Department of Medical Oncology and Pulmonology, University Hospital Tübingen, Otfried-Müller-Str 14, 72076 Tübingen, Germany; DFG Cluster of Excellence 2180 ‘Image-guided and Functionally Instructed Tumor Therapies’ (iFIT), University of Tübingen, Tübingen, Germany

## Abstract

DNA replication is a major source of endogenous DNA damage in tumor cells and a key target of cellular response to genotoxic stress. DNA replication can be deregulated by oncoproteins, such as transcription factor MYC, aberrantly activated in many human cancers. MYC is stringently regulated by the ubiquitin system - for example, ubiquitination controls recruitment of the elongation factor PAF1c, instrumental in MYC activity. Curiously, a key MYC-targeting deubiquitinase USP28 also controls cellular response to DNA damage via the mediator protein 53BP1. USP28 forms stable dimers, but the biological role of USP28 dimerization is unknown. We show here that dimerization limits USP28 activity and restricts recruitment of PAF1c by MYC. Expression of monomeric USP28 stabilizes MYC and promotes PAF1c recruitment, leading to ectopic DNA synthesis and replication-associated DNA damage. USP28 dimerization is stimulated by 53BP1, which selectively binds USP28 dimers. Genotoxic stress diminishes 53BP1–USP28 interaction, promotes disassembly of USP28 dimers and stimulates PAF1c recruitment by MYC. This triggers firing of DNA replication origins during early response to genotoxins and exacerbates DNA damage. We propose that dimerization of USP28 prevents ectopic DNA replication at transcriptionally active chromatin to maintain genome stability.

## Graphical Abstract

**Graphical Abstract:**
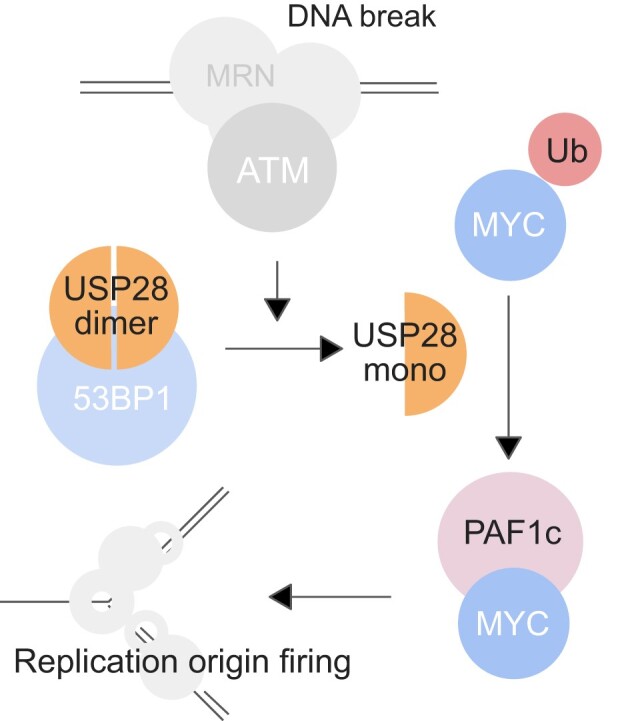

## Introduction

Cellular response to DNA damage is a central element of genome maintenance and tumor suppression. Activated oncogenes can promote genomic instability, leading to tumor heterogeneity and therapy resistance (1). On the other hand, DNA damage is the effector of many cancer therapeutics. Therefore, understanding of mechanisms that induce and respond to DNA damage can be instrumental for the development of effective therapies.

The key element of the DNA damage response (DDR) signaling is the activation of PI3K-related kinases, including ATM and DNAPK (2). DDR-dependent phosphorylation of histones (most prominently, the H2A variant H2AX) and mediator proteins, such as 53BP1, promotes recruitment of DNA repair factors to the DNA lesions (3). In parallel, DDR signaling targets multiple regulators of cell cycle and replication machinery to inhibit DNA synthesis and halt cell cycle progression until the DNA is repaired. Genotoxins typically induce G1 or G2 arrest so that in the latter case cells replicate DNA under stress and arrest only before entering mitosis (4). One critical question is how cells control DNA replication under genotoxic stress and whether these mechanisms can be exploited therapeutically.

A key oncoprotein associated with genomic instability is the transcription factor MYC (also known as cMYC), essential for tumorigenesis in different tissues, including liver, lung, skin and intestine (5–7). MYC is sufficient to induce cell cycle progression and stimulate DNA replication in resting cells, however whether these properties are essential to MYC-driven tumorigenesis is still debated (8,9). Early studies have shown that MYC regulates expression of specific RNAPII-dependent genes via binding to E-box elements in a heterodimeric complex with the Max protein (10–12). Genome-wide chromatin binding data showed that MYC broadly associates with promoters and distal regulatory elements (e.g. enhancers), including sequences that lack consensus E-boxes (13–15). Several studies proposed that MYC amplifies expression of all active genes (16,17), whereas others showed activation and repression of specific groups of genes (18–20).

Whereas MYC-induced changes in gene expression are context-dependent, MYC chromatin binding in most analyzed systems is truly genome-wide (9,16–20), suggesting that some key functions of MYC may be independent of gene expression. For example, MYC was implicated in direct control of DNA replication via interactions with components of the replicative helicase (21–23). Recent studies demonstrated that MYCN, a paralog of MYC in neural tissue, recruits BRCA1 and the exosome complex to promote resolution of R-loops (RNAPII-dependent DNA–RNA hybrids), limit RNAPII collisions with the replisome (transcription–replication conflicts, TRCs) (24,25). Another mechanism involves MYC-mediated recruitment of an elongation factor PAF1c, a multisubunit complex that regulates RNAPII processivity, RNA processing and nuclear export. Besides its role in transcription, PAF1c promotes resolution of TRCs and facilitates DNA repair (26–28). Mechanistically, PAF1c stimulates ubiquitination of histone H2B (29), which in turn stabilizes replication forks and promotes homologous recombination. However, PAF1c can also lead to accumulation of R-loops and stimulate ATR signaling exacerbating replicative stress (30,31), indicating that the function of PAF1c in genome stability depends on genetic or signaling context.

MYC is a short-lived protein and its function is stringently controlled by the ubiquitin system (32,33). Several ubiquitin ligases, including Cul1-based SCF(FBW7) and SCF(Skp2) complexes maintain MYC low protein levels, characteristic of normal untransformed cells. Mutations of these ligases in a subset of human cancers delay MYC degradation (34–37). Intriguingly, ubiquitin conjugation can positively regulate MYC transcriptional function (36,37). For example, ubiquitination (possibly by the Huwe1 ligase) disrupts the inhibitory complex of MYC with PAF1c and promotes transfer of PAF1c on RNAPII (26,27).

Ubiquitination of MYC can be reverted by several deubiquitinases, including USP36 and USP28 (38,39). Curiously, USP28 also plays an important role in cellular response to DNA damage - it stabilizes the key DDR mediator protein 53BP1 and promotes ATM-dependent signaling in response to ionizing radiation (40,41). One intriguing question is how the different facets of USP28 function in regulation of MYC and 53BP1 are integrated in cellular response to DNA damage.

Recent structural and biochemical studies have shown that USP28 forms dimers *in vitro* and in cells (42,43), but the biological role of USP28 dimerization is unknown. Here we show that dimerization of USP28 limits deubiquitination of MYC, thereby controlling interactions with PAF1c. Expression of monomeric USP28 promotes PAF1c recruitment, diminishes TRCs and stimulates DNA replication. We demonstrate that 53BP1 selectively interacts with and stabilizes USP28 dimers, limiting replication-associated DNA double strand breaks. This regulation is disrupted upon genotoxic stress, triggering aberrant DNA synthesis that amplifies DNA damage.

## Materials and methods

### Cell culture and reagents

HLF (Cellosaurus ID CVCL_2947) and p19^−/−^Nras cells (44) were provided by Ramona Rudalska (University Hospital Tübingen). HeLa (Cellosaurus ID CVCL_2947) cells were a gift from Martin Eilers (University of Würzburg). Isolation and immortalization of MEFs was described previously (45). All cell lines were cultured in Dulbecco's modified Eagle's medium (Sigma) with 10% FBS (PAN-Biotech), 1% penicillin–streptomycin (Gibco) and 1% non-essential amino acids (Gibco) in a humidified atmosphere at 37°C and 5% CO_2_. The following reagents and concentrations were used unless otherwise indicated: Etoposide (Cayman, 5 μM), Cycloheximide (Sigma, 100 μg/ml), Thymidine (Sigma, 2 mM), 10074-G5 (Biomol, 10 μM), Topotecan (Sigma, 1 μM), Zeocin (InvivoGen, 100 μg/ml), Cisplatin (Thermo, 10 μM), Gemcitabine (Sigma, 10 μM), Olaparib (Biomol, 10 μM), Simurosertib (MedChemExpress, 2 μM), KU-55933 (Selleckchem, 2 μM), Mirin (Biomol, 25 μM).

### Plasmids, oligonucleotides and antibodies

ORFs encoding USP28-WT or USP28-M were cloned into pRRL-hygro vectors (a gift of Martin Eilers, University of Würzburg). His-Ub plasmids were described previously (46). The sfGFP ORF used to generate USP28-GFP fusion protein was kindly provided by Michael Knop (ZMBH, Heidelberg). For shRNA-mediated silencing, shRNA oligos were cloned into pLKO1.puro (a gift from Bob Weinberg; Addgene plasmid # 8453), shRNAs against hCTR9 (TRCN0000008739/TRCN0000008741) and hCDC73 (TRCN0000008728/TRCN0000011464) in pLKO1.puro were purchased from Sigma. sgRNA-coding oligonucleotides were cloned into the pSpCas9(BB)-2A-Puro (PX459) V2.0 vector (a gift from Feng Zhang; Addgene plasmid # 62988). shRNAs against CDC34A/B in pRetro.Super vector were described previously (46). For USP28-R406Q/ R428T/R510S/R519W, oligonucleotides were cloned into the pcDNA3 vector (45). The sequences of all cloned inserts were confirmed by Sanger sequencing. Oligonucleotide sequences are provided in Supplementary Table S1. Antibody details are shown in Supplementary Table S2.

### Transfection and lentiviral transduction

For transient transfection, PolyJet (SignaGen Laboratories) or Fugene transfection reagent (Promega) were used according to the manufacturer's recommendations.

For CRISPR-Cas9 knockout of *USP28*, Fugene transfection reagent (Promega) was used according to the manufacturer's recommendations. After puromycin selection (0.5 μg/ml), cells were diluted and seeded into a 96-well plate to pick single clones and the knockout was further confirmed by immunoblotting.

For lentiviral transduction, target plasmids were transfected into LentiX cells (a gift of Michael Hudecek, University Hospital Würzburg) together with packaging and envelope plasmids (pPAX2 and pMD2.G, gift from Didier Trono, Addgene plasmid # 12260 & 12259) using polyethylenimine (Sigma). Lentivirus-containing medium was filtered and incubated with target cells for 48–72 h in the presence of 8 μg/ml polybrene (Sigma), followed by antibiotic selection and confirmed by immunoblotting.

### Immunoblotting

Cell pellets were washed with PBS and lysed in TNT-150 buffer (25 mM Tris–HCl pH 7.4, 150 mM NaCl, 1% Triton-X100) supplemented with protease and phosphatase inhibitors (Sigma, 1:1000) on ice for 10 min. Cell lysates were cleared by 10 min centrifugation at 13 000 rpm and 4°C. Equal volume of 4X Laemmli loading buffer (277.8 mM Tris–HCl pH 6.8, 44.4% (v/v) glycerol, 4.4% LDS, 0.02% bromophenol blue, 10% (v/v) beta-mercaptoethanol) was added and lysates were denatured at 95°C for 10 min. Samples were separated on 12% Bis–Tris acrylamide gels, transferred to PVDF membranes, followed by blocking and immunoblotting with target specific antibodies.

For quantification, ImageJ was used to quantify the grayscale intensity of protein bands of interest. The results were further normalized to either loading control (Cycloheximide assay), total protein (DUB activity assay) or reference protein (Immunoprecipitation, His-Ub assay) first and then normalized to the control group for quantification.

### Immunoprecipitation

Cell pellets were washed with PBS and lysed in TNT-250 buffer (25 mM Tris–HCl pH 7.4, 250 mM NaCl, 1% Triton-X100, supplemented with protease and phosphatase inhibitors (Sigma) on ice for 10 min. Cell lysates were cleared by 10 min centrifugation at 13 000 rpm and 4°C. Cleared cell lysates were incubated with antibodies and 30 μl protein of A/G agarose beads (Thermo Scientific, 50% slurry) at 4°C overnight on a rotating wheel. Precipitated complexes were collected by centrifugation and washed three times with the lysis buffer. Samples were denatured by incubation with the 4× Laemmli loading buffer at 95°C for 10 min and analyzed by immunoblotting.

### LC–MS/MS analysis, In-gel digestion

For LC–MS/MS analysis, USP28 and 53BP1 immunoprecipitates were denatured by incubation at 95°C in Laemmli buffer. Proteins were separated on SDS-PAGE gels. Each gel lane was cut into 15 slices. The excised gel bands were destained with 30% acetonitrile in 0.1 M NH4HCO3 (pH 8), shrunk with 100% acetonitrile, and dried in a vacuum concentrator (Concentrator 5301, Eppendorf, Germany). Digests were performed with 0.1 μg trypsin per gel band overnight at 37°C in 0.1 M NH_4_HCO_3_ (pH 8). After removing the supernatant, peptides were extracted from the gel slices with acetonitrile and 5% formic acid, and supernatants of extracted peptides were pooled for each gel slice. NanoLC-MS/MS analyses were performed on an Orbitrap Fusion (Thermo Scientific) equipped with a PicoView Ion Source (New Objective) and coupled to an EASY-nLC 1000 (Thermo Scientific). Peptides were loaded on capillary columns (PicoFrit, 30 cm × 150 μm ID, New Objective) self-packed with ReproSil-Pur 120 C18-AQ, 1.9 μm (Dr Maisch) and separated with a 30-minute linear gradient from 3% to 30% acetonitrile and 0.1% formic acid and a flow rate of 500 nl/min.

Both MS and MS/MS scans were acquired in the Orbitrap analyzer with a resolution of 60 000 for MS scans and 15 000 for MS/MS scans. HCD fragmentation with 35% normalized collision energy was applied. A Top Speed data-dependent MS/MS method with a fixed cycle time of 3 s was used. Dynamic exclusion was applied with a repeat count of 1 and an exclusion duration of 30 s; singly charged precursors were excluded from selection. Minimum signal threshold for precursor selection was set to 50 000. Predictive AGC was used with an AGC target value of 2e5 for MS scans and 5e4 for MS/MS scans. EASY-IC was used for internal calibration.

### MS data analysis

Raw MS data files were analyzed with MaxQuant version 1.6.2.2 (47). Database search was performed with Andromeda, which is integrated in the utilized version of MaxQuant. The search was performed against the UniProt human database (September 2018, UP000005640, 73 099 entries). Additionally, a database containing common contaminants was used. The search was performed with tryptic cleavage specificity with three allowed miscleavages. Protein identification was under control of the false-discovery rate (FDR; <1% FDR on protein and PSM level). In addition to MaxQuant default settings, the search was performed against following variable modifications: Protein N-terminal acetylation, Gln to pyro-Glu formation (N-term. Gln), oxidation (Met), phosphorylation (Ser, Thr, Tyr) and GlyGly (Lys). Carbamidomethyl (Cys) was set as fixed modification. Further data analysis was performed using R scripts developed in-house. Missing LFQ intensities in the control samples were imputed with values close to the baseline. Data imputation was performed with values from a standard normal distribution with a mean of the 5% quantile of the combined log_10_-transformed LFQ intensities and a standard deviation of 0.1. For the identification of significantly enriched proteins, boxplot outliers were identified in intensity bins of at least 300 proteins. Log_2_ transformed protein ratios of sample versus control with values outside a 1.5× (significance 1) or 3× (significance 2) interquartile range (IQR), respectively, were considered as significantly enriched. The proteomic data are deposited at the PRIDE database (submission #616633).

### Assessment of cell proliferation

100 000 cells of each tested cell line were seeded per well of a 6-well plate and cultured until one well was close to confluency. Cells were washed with PBS and fixed with 1% PFA for 10 min at room temperature, PFA was removed and 2 ml of crystal violet solution (Sigma) was added into each well and incubated at room temperature for 10 min. Fixed cells were washed three times with 2 ml PBS to remove the remaining crystal violet solution and wells were scanned for quantification.

### Colony formation assay

5000 cells of each tested cell line were mixed well with low melting point agarose (final concentration 0.25%) in 1 ml DMEM medium and seeded into one well of a 6-well plate (precoated with 1.5 ml 1% regular agarose) and cultured for 7 days until colonies were formed.

### Immunofluorescence

Cells were cultured on 10 mm round glass slides in 6-well plates, fixed with 1% PFA in PBS for 10 min at room temperature, permeabilized/blocked with 1% BSA (Sigma, in TBST) for 20 min. Primary antibody was added (1:100–1:1000) and incubated at room temperature for 1–4 h, then secondary antibody was added (1:100) after three times PBS washing and incubated at room temperature for 1–4 h. Slides were further mounted on coverslips with a DAPI-containing mounting solution, sealed by nail polish and stored at 4°C in the dark.

### EdU/EU incorporation

Cells were cultured on 10 mm round glass slides in 6-well plates and treated with EdU (25 μM) or EU (10 μM) for 20 min before fixing with 1% PFA for 10 min at room temperature and permeabilized with 0.3% Triton-X100 (in TBS) for 10 min. Click reaction was carried out by addition of 2 mM CuSO_4_, 0.4 μM Sulfo-Cy3-azide, 100 mM Na Ascorbate (in PBS) and incubated at room temperature for 30 min in the dark, then washed three times with PBS and further mounted on glass slides with a DAPI-containing mounting solution and sealed by nail polish and stored at 4°C in the dark.

### Proximity-ligation assays

Cells were cultured on 10 mm round glass slides in 6-well plates and fixed with 1% PFA for 10 min at room temperature. Cells were permeabilized with 0.3% Triton-X100 (in TBS) for 20 min and blocked by 2.5% BSA in TBST. The assays were performed with Duolink® in Situ Detection Reagents Red or Green Kit according to the manufacturer's instructions (Sigma). For PLA with antibodies against biotin, click reaction was done as described in the ‘EdU/EU incorporation’ section (with biotin-azide instead of Sulfo-Cy3-azide) prior to incubation with antibodies.

### Real-time quantitative PCR

Total RNA was extracted with TRIzol reagent (Sigma) according to the manufacturer's protocol and DNase I (NEB) was added to remove remaining DNA. cDNA was reversed with M-MLV Reverse Transcriptase (Promega) according to the manufacturer's protocol. The expression level of target mRNA was normalized to the expression level of β-Actin. Primer sequences are provided in Supplementary Table S1.

### DUB activity assays

Cell pellets collected form 6-well plate were washed by PBS and lysed in 200 μl 1% Triton-X100 in PBS, with 1:1000 protease and phosphatase inhibitors on ice for 5 min and centrifuged at 1000 g and 4°C for 5 min. 70 μl supernatant was incubated with 5 μl PBS with or without 0.25 μg concentrated probe (VS/VME-Ubiquitin, UbiQ) at room temperature for 5 min. The reaction was terminated and proteins were denatured by addition of 4× Laemmli loading buffer and incubation at 95°C for 10 min and analyzed by immunoblotting.

### Ubiquitin pulldown assays

Expression vectors for MYC, His-tagged ubiquitin and USP28 variants were transfected into HeLa cell lines with PolyJet transfection reagent (SignaGen Laboratories). Twelve hours after transfection, the cell culture medium was replaced. Forty-eight hours after transfection cells were collected and lysed in 1 ml urea buffer (8 M urea, 10 mM Imidazole in PBS) at room temperature. Lysates were briefly sonified and cleared by centrifugation at 13 000 rpm for 10 min at room temperature. Supernatants were further incubated with 20 μl Ni-NTA beads (Cube Biotech, 50% slurry) at room temperature overnight with rotation. Beads were centrifuged and washed twice with Urea Buffer, denatured with 4X Laemmli loading buffer at 95°C for 10 min before separation on Bis–Tris gels and immunoblotting.

### Nascent chromatin capture assays

The nascent chromatin capture assay was performed as described previously (48) with slight modifications. Cells were treated with a mixture of biotin-16-dUTP and biotin-16-dCTP (0.5 μM each, Jena Bioscience) in hypotonic buffer (50 mM KCl, 10 mM HEPES) for 5 min and followed by another 5 min dUTP/dCTP treatment in regular DMEM medium (For etoposide treated samples, etoposide was added 20 min prior dUTP/dCTP treatment and kept until cells were fixed). Cells were then fixed by 0.2% PFA for 5 min at room temperature and quenched by co-incubation with 200 mM glycine for 1 min. Cells were resuspended in TNT-300 buffer (25 mM Tris–HCl pH 7.4, 300 mM NaCl, 1% Triton-X100) together with protease and phosphatase inhibitors (Sigma, 1:1000) and sonified (30% amplitude, 45 s on/15 s off for 10 min) before incubated with 10 μl of Streptavidin Magnetic Beads (50% slurry, NEB) at room temperature 45 min on a rotating wheel. Beads were collected by centrifugation and washed three times with the TNT-300 buffer. Samples were denatured by incubation with the 4× Laemmli loading buffer at 95°C for 10 min and analyzed by immunoblotting.

### DNA fiber assays

The fiber assay was performed as described previously (49). Briefly, cells were incubated each 20 min with 25 μM IdU and subsequently with 250 μM CldU at 37°C. Cells are resuspended in PBS after harvesting and transferred on a coverslip, lysis solution (200 mM Tris pH 7.5, 50 mM EDTA, 0.5% SDS) was added and the slides were air dried in an angle to allow DNA to spread over the slide. DNA was fixed with pre-chilled Methanol: Acetic Acid (2:1) before incubation with 2.5 M HCl for 100 min. The slides were blocked and the DNA fibers were stained with antibodies against IdU and CldU. For quantification, DNA fiber lengths were measured by ImageJ and converted to fork velocity using the following formula: 1 μm = 2.59 kb.

### Neutral comet assays

Cells were detached by Trypsin-EDTA (Life Technologies) and pellets (containing 10 000–20 000 cells) were washed with PBS and resuspended in 200 μl of 0.7% LMP agarose to obtain single-cell suspensions. 65 μl of the mixture was dropped and covered by a coverslip on the glass slide which pre-coated by 0.8% regular agarose. After solidification, another 80 μl of 0.7% LMP agarose was added to cover as the top layer. Cells were lysed in lysis solution (2.5 M NaCl, 0.1 M EDTA, 10 mM Trizma base pH = 10, 1% N-laurylsarcosine, 0.5% Triton X-100, 10% DMSO final) in dark at 4°C overnight and electrophoresis was preceded in TAE buffer with 0.5 V/cm for 1 h. Then cells were fixed by absolute ethanol and stained by ethidium bromide (2 μg/ml in water) for microscopy.

### Cut & run

Cut&Run assay was performed on PFA-fixed cells using the CUT&RUN assay kit (CST) according to the manufacturer's recommendations. In brief, 1 million of 0.2% PFA fixed cells were washed with 1 ml C & R Wash buffer (20 mM HEPES pH 7.5, 150 mM NaCl, 0.5 mM Spermidine, Protease/Phosphatase inhibitors 1:1000) and 2% were kept as input. The rest were incubated with 40 μl of concanavalin A-coated beads (G biosciences, prewashed and resuspended in C & R Binding buffer (20 mM HEPES pH 7.5, 10 mM KCl, 1 mM CaCl_2_, 1 mM MnCl2)) at room temperature for 10 min with rotation. Samples were washed by Wash buffer and pellets were incubated with antibodies against LEO1 or IgG in 150 μl C & R Antibody buffer (C & R TritonX-Wash buffer and 2 mM EDTA) at 4°C in a shaker with 800 rpm overnight. Subsequently, beads were washed with C & R TritonX-Wash buffer (C & R Wash buffer with 0.1% TritonX-100) and incubated with Protein-A/G-MNase (1:2000, CST) in 150 μl C & R TritonX-Wash buffer at 4°C with 800 rpm for 1 h. Beads were further washed with TritonX-Wash buffer and Low-Salt Rinse buffer (20 mM HEPES pH 7.5, 0.5 mM Spermidine, 0.1% TritonX-100) before incubated in 200 μl C & R Incubation buffer (3.5 mM HEPES pH 7.5, 10 mM CaCl_2_, 0.1% TritonX-100) at 0°C for 30 min. Afterwards, beads were incubated in 200 μl C & R Stop buffer (170 mM NaCl, 20 mM EGTA, 0.1% TritonX-100, 50 μg/ml RNase A) at 37°C for 30 min to release DNA fragments. Supernatant was incubated at 55°C overnight with 2 ul 10% SDS and 5 ul proteinase K followed by phenol/chloroform/isoamyl extraction. DNA was further precipitated in absolute ethanol and GlycoBlue at –20°C overnight and dissolved in the water for qPCR. For input samples, they were sonicated in 200 μl TE buffer (10 mM Tris–HCl pH 8.0, 1 mM EDTA) with 250 mM NaCl and 0.5% SDS at 4°C with the following parameters to obtain the fragmented DNA: 100% cycle, 30% amplitude, 45 sec on/15 sec off for 10 min. Subsequently, samples were incubated with 1 μl of RNase A at 50°C for 2 h and then mixed with 2 μl of proteinase K in the same buffer and incubated at 65°C overnight. Afterwards, samples were extracted and precipitated as described above for qPCR.

### RNA sequencing

Total RNA was extracted with TRIzol reagent (Sigma) according to the manufacturer's protocol and DNase I (NEB) was added to remove remaining DNA. 1 μg of purified RNA was processed according to the manufacture protocol of the NEBNext® Ultra™ RNA Library Prep Kit for Illumina® (E7530) and with the NEBNext® Multiplex Oligos for Illumina® (Dual Index Primer Set 1) (E7600). Libraries were sequenced on the Illumina Novaseq 6000 instrument. Mapping of fastq files was performed with STAR (50) and differentially expressed genes were identified using EdgeR (51). The RNA sequencing data discussed in this publication have been deposited in NCBI’s Gene Expression Omnibus and are accessible through GEO Series accession number GSE213892 (https://www.ncbi.nlm.nih.gov/geo/query/acc.cgi?acc=GSE213892).

### Image analysis

Images were analysed automatically with the free software FIJI/ImageJ version 1.53f (https://imagej.net/software/fiji/). In brief, images were segmented based on nuclear areas in the DAPI channel, afterwards, staining intensity in other channels (Immunofluorescence assays and EdU/EU incorporation assays) or the number of foci (PLA assays) was measured for each nucleus. For PLAs, several z-layers were combined to a single image by maximum intensity projection prior to counting the number of PLA foci using the ‘Find Maxima’ command. For neutral comet assays, the free plugin ‘OpenComet’ (https://cometbio.org/) was used.

### Statistical analysis

Statistical analysis was done using GraphPad Prism 9 (GraphPad Software Inc.). For comparison between two groups, two-tailed, unpaired t tests or Mann-Whitney tests were used depending on the normal distribution of data; For comparison between multiple groups, ordinary one-way ANOVA followed by the suitable post-hoc test or the Kruskal–Wallis test were used depending on the normal distribution of data. Non-linear fit model-one phase decay was used for protein half-life determination. Linear regression was used for correlation analysis. Sample sizes and *P*-values are shown in the figure legends.

## Results

### Dimerization of USP28 controls MYC turnover

**Figure 1.**
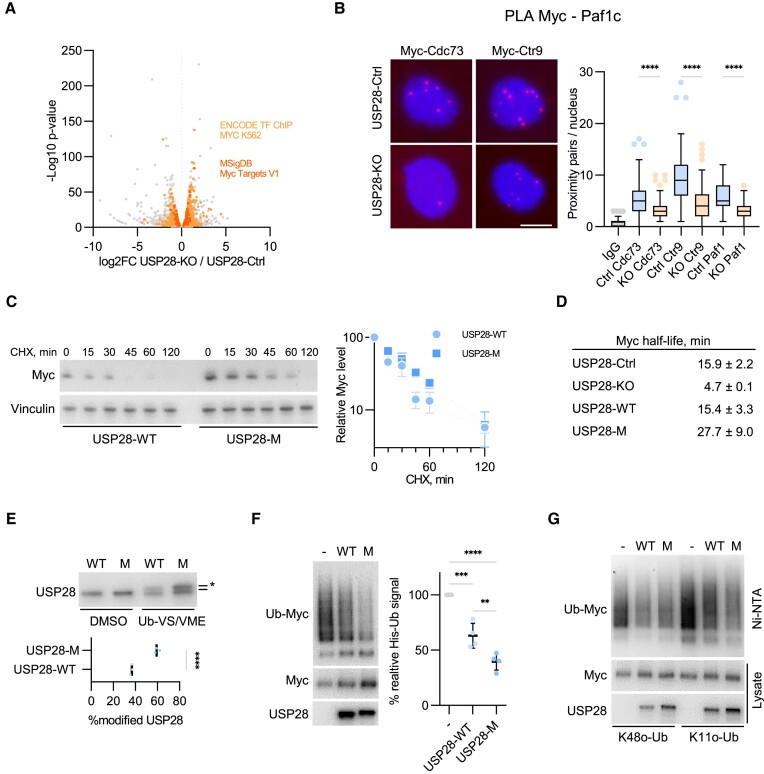
Dimerization of USP28 controls MYC stability and interaction with PAF1c. **(A)** RNA-seq analysis of gene expression in HLF USP28-KO cells compared to HLF Control (USP28-Ctrl) cells. Highlighted are the top enriched sets for the Encode TF ChIP and MsigDB datasets, based on the analysis by the Enrichr portal (98). See also Supplementary Figure S1D. **(B)** PLA assays with antibodies against MYC and PAF1c subunits (CDC73/CTR9/PAF1) or non-specific IgG control antibody (IgG) in HLF USP28-Ctrl/KO cells. Quantification shows data points for one representative experiment from three independent biological replicates (*n* = 3). At least 71 cells were quantified. The data were analyzed with Kruskal–Wallis test followed by Dunn's multiple comparison of selected pairs, ****P < 0.0001. Scale bar = 10 μm. **(C)** Immunoblotting analysis of USP28-KO HLF cells, expressing USP28-WT or USP28-M, treated with cycloheximide (100 μg/ml) for the indicated time points. Image shows one representative experiment from three independent biological replicates (*n* = 3). MYC protein half-life was determined by a non-linear fit model. Right panel shows the mean of the three independent biological replicates. Error bars denote S.D. **(D)** The half-life of MYC in HLF USP28-Ctrl, USP28-KO or USP28-KO cells reconstituted with USP28-WT or USP28-M variants, calculated from data shown in panel (C), Figure S1B and replicate experiments. **(E)** DUB activity assays in whole cell lysates of HLF cells, expressing USP28-WT or USP28-M. Image shows one representative experiment (*n* = 3). The asterisk shows the Ub-VS/VME modified USP28. Lower panel shows the mean of the three independent biological replicates (*n* = 3). The data were analyzed with two-tailed, unpaired *t* test, ****P < 0.0001. **(F)** Ubiquitin pulldown assays with HeLa USP28-KO cells, expressing MYC, WT His-Ub and USP28-WT/M. Image shows one representative experiment (*n* = 4). Right panel shows the mean of the four independent biological replicates. The data were analyzed with ordinary one-way ANOVA followed by Tukey's multiple comparison test of selected pairs, **P < 0.01, ***P < 0.001, ****P < 0.0001. **(G)** Ubiquitin pulldown assays with HeLa USP28-KO cells expressing MYC, K48-only or K11-only His-Ub and USP28-WT/M. Image shows one representative experiment (*n* = 2).

Ubiquitination of MYC controls its transcriptional function, in part, by disrupting a complex of MYC with the elongation factor PAF1c (27). Loss of USP28 promotes MYC ubiquitination (45), predicting a diminished MYC-PAF1 interaction and enhanced expression of MYC target genes in USP28-deficient cells. To test this hypothesis, we used CRISPR to delete the USP28 gene in a hepatocellular carcinoma (HCC) cell line HLF (Supplementary Figure S1A). Deletion of USP28 downregulated MYC protein, whereas knockout of a related DUB USP25 did not affect MYC levels (Supplementary Figure S1B). Cycloheximide assays confirmed that USP28 knockout decreased MYC protein stability (Supplementary Figure S1C). RNA-seq analysis showed that deletion of USP28 deregulated MYC-dependent transcription—among 4205 deregulated genes, known MYC-bound and -regulated genes were the top enriched groups (Figure 1A; Supplementary Figure S1D). This was accompanied by a pronounced reduction of MYC association with PAF1c subunits CDC73, CTR9 and PAF1 (Figure 1B), in line with previous observations (27).

In vitro and in cells USP28 forms homodimers, which can be disrupted by a single point mutation of a critical leucine within the dimerization interface (L545E) (42,43). To analyze the impact of USP28 dimerization on regulation of MYC, we reconstituted USP28-deficient HLF cells with HA-tagged wildtype (USP28-WT) or monomeric (USP28-M) USP28 alleles using lentiviral transduction. Both USP28 variants localized to the nucleus (Supplementary Figure S1E) and interacted with endogenous MYC, as determined by PLA assays with antibodies against USP28 and MYC (Supplementary Figure S1F). Compared to wildtype USP28, monomeric USP28 had a stronger stabilizing effect on MYC in HLF cells and in a p19Arf-deficient, Nras-transformed murine HCC cell line established from an autochthonous tumor (p19-/-Nras) (44). Similar results were obtained in mouse embryonic fibroblasts (MEFs) and HeLa cells (Figure 1C and D; Supplementary Figure S1, G and H). Another USP28 substrate Jun was also stabilized by USP28-M (Supplementary Figure S1H), indicating that dimerization can blunt the catalytic activity of USP28 toward different substrates in various cellular contexts. In line with this view, incubation of lysates of cells, expressing USP28-WT or USP28-M with DUB-reactive probes Ub-VME and Ub-VS (52,53), revealed a more complete conversion into the Ub-modified form for the monomeric USP28 (Figure 1E).

Ubiquitin pulldown assays in USP28-KO HeLa cells (45) transfected with vectors expressing MYC, His-tagged ubiquitin and USP28 variants, showed that USP28-M more potently promoted deubiquitination of MYC compared to wildtype USP28 (Figure 1F; Supplementary Figure S1I). Since USP28 can disassemble degradative K48- and K11-linked ubiquitin chains (53,54), we compared the activity of USP28 using pulldown assays with His-Ub variants bearing K48 or K11 as a sole internal ubiquitin acceptor. USP28-M diminished MYC-Ub signal for K11-linked chains stronger than USP28-WT, whereas MYC-conjugated K48 chains were reduced similarly by both variants (Figure 1G). To control for the contribution of endogenous ubiquitin in these assay, we knocked down CDC34, the E2 enzyme that acts with the FBW7(SCF) E3 complex to assemble K48 chains on MYC (46,55), using two shRNAs against CDC34A and CDC34B. Depletion of CDC34 reduced the effect of USP28-WT on MYC-conjugated K11 chains, whereas USP28-M still efficiently deubiquitinated MYC (Supplementary Figure S1J). We concluded that dimerization of USP28 restricts its activity, primarily towards K11-linked ubiquitin chains.

### USP28 monomers stimulate DNA replication

**Figure 2.**
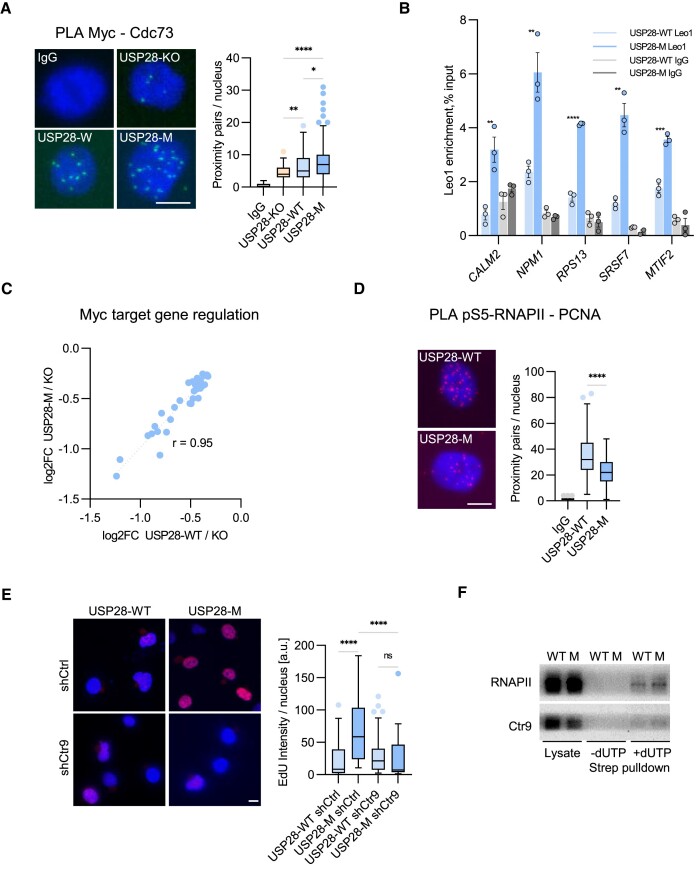
USP28 dimerization limits PAF1c recruitment to chromatin and restricts DNA replication. **(A)** PLA assays with antibodies against MYC, CDC73 or IgG in HLF USP28-KO cells expressing Usp28-WT/M or a control vector. Quantification shows data points for one representative experiment (*n* = 2). At least 67 cells were quantified. The data were analyzed with Kruskal-Wallis test followed by Dunn's multiple comparison of selected pairs, *P < 0.05, **P < 0.01, ****P < 0.0001. Scale bar = 10 μm. **(B)** Cut&Run assay followed by qPCR analysis showing PAF1c subunit LEO1 abundance on MYC target promoters in HLF USP28-KO cells expressing USP28-WT or USP28-M. Quantification shows data points for one representative experiment (*n* = 2). The data were analyzed from three technical replicates with two-tailed, unpaired *t* test for each pair, **P < 0.01, ***P < 0.001, ****P < 0.0001. **(C)** Regulation (log2FC) of a subset of MYC target genes (MsigDB Hallmark set MYC targets V1) in HLF USP28-KO cells, expressing either USP28-WT or USP28-M. The Pearson correlation coefficient r equals 0.95. **(D)** PLA assays with antibodies against pS5-RNAPII and PCNA or a control IgG in HLF USP28-KO cells, expressing USP28-WT/M. Quantification shows data points for one representative experiment (*n* = 3). At least 95 cells were quantified. The data were analyzed with two-tailed, Mann–Whitney test, ****P < 0.0001. Scale bar = 10 μm. **(E)** EdU incorporation assays in HLF USP28-WT or USP28-M cells, expressing shCtrl/shCTR9. At least 72 cells were quantified. Quantification shows data points for one representative experiment (*n* = 3). The data were analyzed with Kruskal–Wallis test followed by Dunn's multiple comparison of selected pairs, ****P < 0.0001, ns *P* > 0.05. Scale bar = 10 μm. **(F)** Immunoblotting analysis of protein levels of RNAPII and CTR9 on nascent chromatin, captured from lysates of HLF USP28-WT or USP28-M cells. Image shows one representative experiment (*n* = 2).

Consistent with effects on MYC turnover, PLA experiments showed that MYC-PAF1c interaction was potentiated in cells expressing USP28-M compared to USP28-WT (Figure 2A, Supplementary Figure S2A). Cut&Run assays with antibodies against a PAF1c subunit LEO1 showed an increased recruitment to MYC target promoters in cells expressing USP28-M compared to USP28-WT (Figure 2B). Unexpectedly, transcriptome profiling showed that expression of USP28-WT and USP28-M in USP28-KO cells equally regulated expression of MYC target genes (Figure 2C). In contrast, the impact of USP28-WT and USP28-M on colony formation, both under standard growth conditions and in soft agar, was clearly different (albeit variable in different cell lines, Supplementary Figure S2, B, C and D), suggesting that ectopic stabilization of MYC by USP28-M has largely non-transcriptional effects.

Both MYC and PAF1c have transcription-independent functions in DNA replication (21,22,28). In particular, PAF1c facilitates resolution of TRCs and promotes DNA replication under stress (28). PLA assays with antibodies against pS5-RNAPII and PCNA to assess TRCs (56) showed that expression of USP28-M reduced the incidence of conflicts compared to USP28-WT (Figure 2D). Furthermore, cells expressing USP28-M showed an enhanced rate of EdU incorporation compared to USP28-WT cells (Figure 2E), indicative of accelerated DNA replication. Nascent chromatin capture assays (48) showed an increased enrichment of RNAPII and CTR9 on nascent DNA in USP28-M cells (Figure 2F), suggesting that monomeric USP28 stimulates DNA replication in the vicinity of RNAPII/PAF1c-bound sites. Depletion of CTR9 abolished the increase in EdU incorporation in USP28-M cells (Figure 2E; Supplementary Figure S2E), arguing that USP28-M stimulated DNA replication via PAF1c.

### Monomeric USP28 induces replication-dependent DNA damage

**Figure 3.**
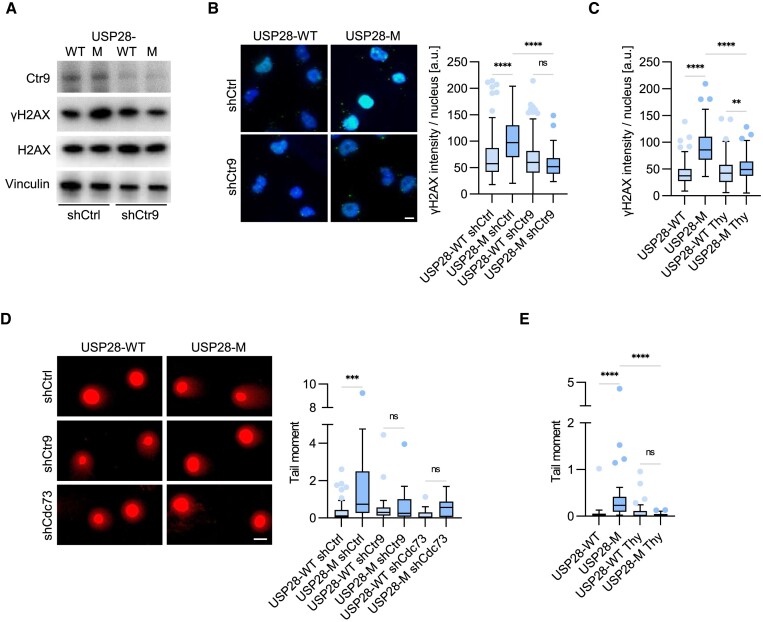
Monomeric USP28 induces replication-dependent DNA damage. **(A)** Immunoblot analysis documenting γH2AX levels in HLF USP28-WT or USP28-M cells with shCtrl/shCTR9. Image shows one representative experiment (*n* = 3). **(B)** Immunofluorescence analysis documenting γH2AX signal intensity in HLF USP28-WT or USP28-M cells, expressing shCtrl or shCTR9. Quantification shows data points from one representative experiment (*n* = 3). At least 122 cells were quantified. The data were analyzed with Kruskal-Wallis test followed by Dunn's multiple comparison of selected pairs, ****P < 0.0001, ns *P* > 0.05. Scale bar = 10 μm. **(C)** Immunofluorescence analysis with γH2AX antibodies in USP28-WT or USP28-M cells treated with thymidine (2 mM) or vehicle control for 2 h. Quantification shows data points for one representative experiment (*n* = 3). At least 155 cells were quantified. The data were analyzed with Kruskal–Wallis test followed by Dunn's multiple comparison of selected pairs, **P < 0.01, ****P < 0.0001. Scale bar = 10 μm. **(D)** Neutral comet assays in USP28-WT or USP28-M cells with shCtrl/shCTR9/shCDC73. Quantification shows data points for one representative experiment (*n* = 3). At least 18 cells were quantified. The data were analyzed with Kruskal–Wallis test followed by Dunn's multiple comparison of selected pairs, ***P < 0.001, ns *P* > 0.05. Scale bar = 10 μm. **(E)** Neutral comet assays in USP28-WT or USP28-M cells treated with thymidine (2 mM) or vehicle control for 2 h. Quantification shows data points for one representative experiment (*n* = 3). At least 27 cells were quantified. The data were analyzed with Kruskal–Wallis test followed by Dunn's multiple comparison of selected pairs, ****P < 0.0001, ns *P* > 0.05.

Deregulated DNA replication can lead to genomic instability (57,58). We therefore compared levels of γH2AX, a marker of DNA damage, in cells expressing USP28-WT and USP28-M using immunoblotting and immunofluorescence assays. Expression of USP28-M upregulated γH2AX levels compared to USP28-WT (Figure 3A and B; Supplementary Figure S3A and B). Knockdown of PAF1c decreased γH2AX levels, in agreement with the effects on DNA replication (Figure 3A and B; Supplementary Figure S3, A and B). Importantly, addition of excess thymidine, which inhibits ribonucleotide reductase and blocks DNA synthesis (59), diminished the increase in γH2AX in cells expressing USP28-M (Figure 3C). Arrest of cell cycle by serum deprivation also decreased and equalized γH2AX levels in both cell lines (Supplementary Figure S3C), suggesting that DNA damage induced by monomeric USP28 requires DNA replication.

Assessment of DNA breakage using neutral comet assay (60) showed an increased level of DSBs in cells expressing USP28-M compared to USP28-WT (Figure 3D). Depletion of PAF1c subunits reverted this effect (Figure 3D), indicating that ectopic recruitment of PAF1 underlies the induction of DNA damage in cells expressing USP28-M. As for γH2AX levels, addition of thymidine diminished comet tail length in USP28-M cells (Figure 3E), arguing that DNA damage in these cells depends on DNA replication.

USP28 mutations within the dimerization domain occur in a number of human cancers (61) and potentially may affect dimer formation. To test the impact of several such mutations on USP28 dimerization, we expressed FLAG-tagged USP28-WT and HA-tagged USP28 wildtype or mutant alleles and performed immunoprecipitation with HA antibodies. The analyzed mutants, especially R519W, showed a reduced ability to form dimers (Supplementary Figure S3D). This result was confirmed in PLA experiments with FLAG and HA-tag antibodies (Supplementary Figure S3E). Importantly, expression of the R519W variant upregulated γH2AX levels (Supplementary Figure S3F), indicating that cancer-associated mutations can impair USP28 dimerization and contribute to genomic instability in tumor cells.

### DDR signaling diminishes USP28 dimerization

**Figure 4.**
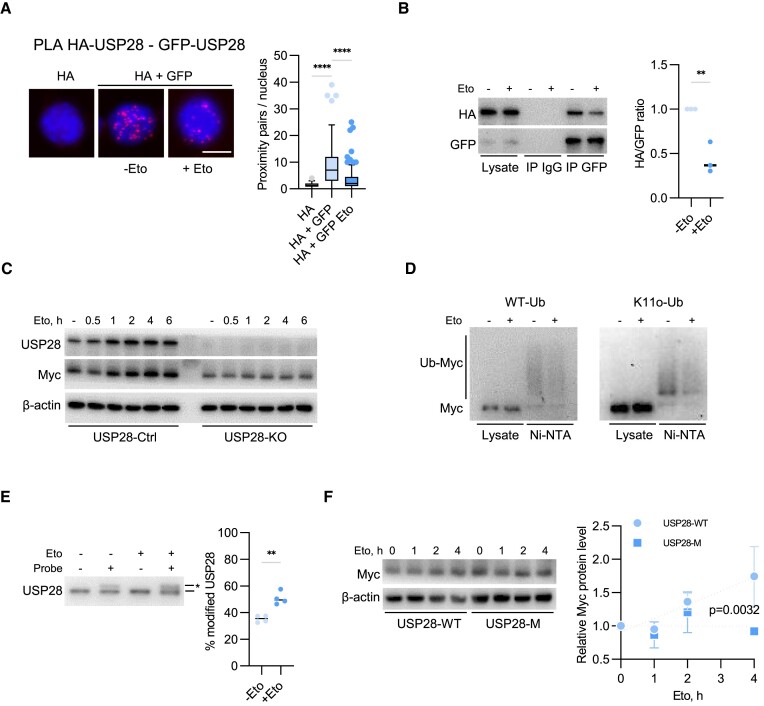
DNA damage diminishes USP28 dimerization. **(A)** PLA assays with antibodies against GFP and HA-tag in p19^−/−^Nras cells, expressing GFP- and HA-tagged USP28 with or without etoposide (5 μM, 30 min) treatment. Quantification shows data points for one representative experiment (*n* = 3). At least 26 cells were quantified. The data were analyzed with Kruskal-Wallis test followed by Dunn's multiple comparison of selected pairs, ****P < 0.0001. Scale bar = 10 μm. **(B)** Immunoprecipitation analysis with the GFP antibodies in p19^−/−^Nras cells expressing HA and GFP-tagged USP28, treated with DMSO or etoposide (5 μM, 30 min). Right panel shows the mean of three independent biological replicates (*n* = 3). The data were analyzed with two-tailed, unpaired *t* test, **P < 0.01. **(C)** Immunoblotting analysis of HLF USP28-Ctrl/KO cells treated with etoposide (5 μM) for the indicated time points. Image shows one representative experiment (*n* = 2). **(D)** His-Ub pulldown assay in HeLa cells transfected with MYC and WT or K11-only His-Ub before and after etoposide (5 μM, 30 min) treatment. Image shows one representative experiment (*n* = 3). **(E)** DUB activity assay in HLF USP28-Ctrl cells with or without etoposide (5 μM, 30 min) treatment. The asterisk shows the Ub-VS/VME modified USP28. Right panel shows the mean of four independent biological replicates (n = 4). The data were analyzed with two-tailed, unpaired *t* test, **P < 0.01. **(F)** Immunoblots documenting MYC protein level in HLF USP28-WT/M cells, treated with etoposide (5 μM) for the indicated time points. Right panel shows the mean of three independent biological replicates (*n* = 3). Linear regression analysis shows that the slopes of the regression lines differ significantly (*P* = 0.0032). Error bars denote S.D.

Previous work has shown that the USP28 catalytic function is activated by DDR signaling (40,41,62), leading us to hypothesize that formation of USP28 monomers can underlie USP28 activation during DDR. To test this idea, we generated p19^−/−^Nras cell lines, stably expressing HA- and GFP-tagged USP28 proteins (Supplementary Figure S4A) and analyzed interaction of these proteins before and after treatment with etoposide, which increases MYC protein levels (63). PLA assays with HA and GFP antibodies showed a robust signal in unstressed cells, which was diminished after treatment with etoposide or other genotoxins (Figure 4A; Supplementary Figure S4B), suggesting that disruption of USP28 dimers is a common event in DDR. This view was supported by immunoprecipitation assays (Figure 4B, Supplementary Figure S4C) and by native gel electrophoresis of lysates of HeLa cells, transfected with wildtype USP28 (Supplementary Figure S4D). Since USP28 is a target of ATM (41), the apical DDR kinase, we tested the impact of ATM inhibition on USP28 dimers. Treatment with KU-55933, a specific ATM inhibitor, prevented the disassembly of USP28 dimers, induced by etoposide (Supplementary Figure S4E, compare to Supplementary Figure S4B), suggesting that activation of ATM upon genotoxic stress leads to formation of Usp28 monomers.

Treatment with etoposide led to the accumulation of MYC protein in HLF and p19^−/−^Nras cells (Figure 4C, Supplementary Figure S4F), whereas MYC mRNA was not significantly affected, ruling out transcriptional regulation (Supplementary Figure S4G). MYC levels did not increase in USP28-KO cells, arguing that MYC is stabilized in a USP28-dependent manner in response to etoposide (Figure 4C). Ubiquitin pulldown assays in HeLa cells showed a decrease in MYC ubiquitination in etoposide-treated cells compared to DMSO-treated cells (Figure 4D). Knockout of USP28 abolished this effect, demonstrating that etoposide reduces MYC ubiquitination via USP28 (Supplementary Figure S4H). In accord, incubation of total cell lysates with DUB-reactive probes revealed a higher activity of USP28 after etoposide treatment (Figure 4E). Etoposide did not increase MYC abundance in cells expressing monomeric USP28, which had elevated basal MYC levels (Figure 4F; Supplementary Figure S4F), demonstrating that stabilization of MYC upon etoposide treatment involves formation of USP28 monomers.

### 53BP1 controls USP28 dimerization and catalytic activity

**Figure 5.**
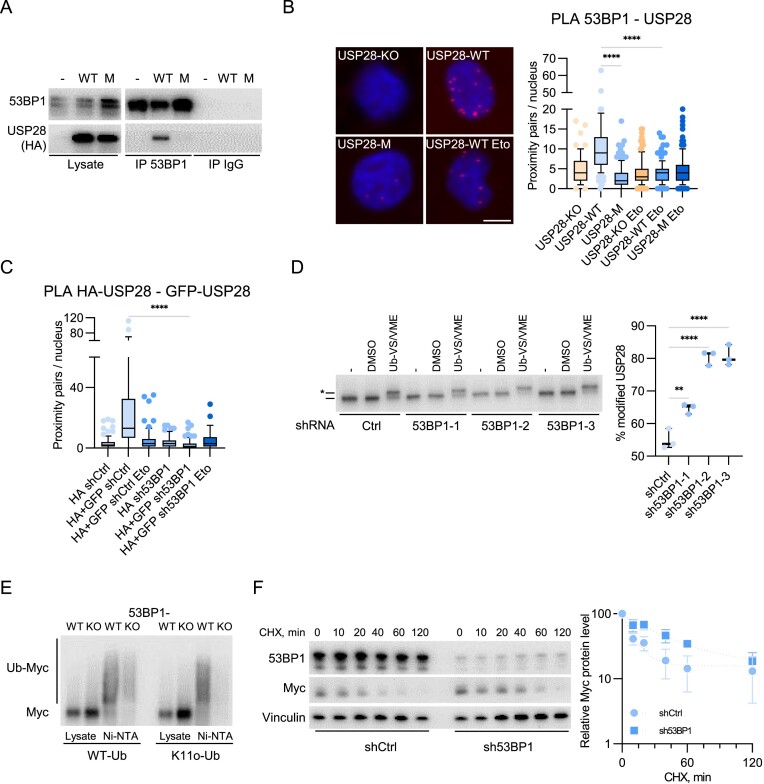
53BP1 controls USP28 dimerization and catalytic activity. **(A)** Immunoprecipitation analysis with antibodies against 53BP1 or control IgG from HLF cells expressing wildtype (WT) or monomeric (M) USP28. Image shows one representative experiment (*n* = 3). **(B)** PLA assays with antibodies against 53BP1 and HA-tag in HLF USP28-KO/WT/M cells, treated with DMSO or etoposide (5 μM, 30 min). Quantification shows data points for one representative experiment (*n* = 3). At least 71 cells were quantified. The data were analyzed with Kruskal–Wallis test followed by Dunn's multiple comparison of selected pairs, ****P < 0.0001. **(C)** PLA assays with antibodies against GFP and HA-tag in p19^−/−^Nras cells with shCtrl/sh53BP1 treated with DMSO or etoposide (5 μM, 30 min). Quantification shows data points for one representative experiment (*n* = 3). At least 42 cells were quantified. The data were analyzed with Kruskal-Wallis test followed by Dunn's multiple comparison of selected pair, ****P < 0.0001. **(D)** DUB activity assay in HLF sh53BP1 cells. The asterisk shows the Ub-VS/VME modified USP28. Right panel shows the mean of three independent biological replicates (*n* = 3). The data were analyzed with ordinary one-way ANOVA test followed by Tukey's multiple comparison of selected pairs, **P < 0.01, ****P < 0.0001. **(E)** His-Ub pulldown assay in HeLa cells, transfected with MYC, WT-Ub or K11-only His-Ub showing the deubiquitination of MYC with or without 53BP1 knockout. Image shows one representative experiment (*n* = 3). **(F)** Immunoblotting analysis of MYC protein level in HLF shCtrl/sh53BP1 cells treated with cycloheximide (100 μg/ml) for the indicated time points. Right panel shows the mean of three independent biological replicates (*n* = 3). Error bars denote S.D.

A major USP28 binding partner is 53BP1, which can recruit USP28 to the sites of DNA damage (41,64). Our mass spectrometry analysis and followup immunoprecipitation assays showed that USP28 interacts with 53BP1 in unstressed cells (Supplementary Figure S5A and B), suggesting a role for the 53BP1–USP28 complex during unperturbed cell cycle (41,65). Strikingly, PLA and immunoprecipitation assays showed that 53BP1 selectively binds wildtype but not monomeric USP28 (Figure 5A and B; Supplementary Figure S5C). Etoposide treatment diminished 53BP1-USP28 interaction in an ATM-dependent manner (Figure 5B; Supplementary Figure S5D), correlating with the disruption of USP28 dimers. Depletion of 53BP1 in p19^−/−^Nras cells diminished USP28 dimerization (Figure 5C; Supplementary Figure S5E), mimicking the effect of etoposide. ATM inhibition increased USP28 dimerization in etoposide-treated shCtrl cells but not in sh53BP1 cells (Supplementary Figure S5F), indicating that interaction with 53BP1 can stabilize USP28 dimers and that this is antagonized by ATM.

Since USP28 monomers are more active towards MYC, we compared USP28 catalytic activity in HLF cells expressing shCtrl and sh53BP1. Incubation of total cell lysates with the Ub-VME/Ub-VS probes showed that depletion of 53BP1 stimulated USP28 deubiquitinase activity (Figure 5D, Supplementary Figure S5G), whereas the abundance of USP28 was not changed (Supplementary Figure S5H and I). His-tagged ubiquitin pulldown assays in 53BP1-knockout HeLa cells (Supplementary Figure S5H and I) with either wildtype or K11-only ubiquitin revealed a reduced MYC ubiquitination in 53BP1-knockout HeLa cells (Figure 5E). In accord, loss of 53BP1 increased steady state MYC levels and stabilized MYC protein whereas MYC mRNA levels were slightly reduced (Figure 5F; Supplementary Figure S5H and J). We concluded that depletion of 53BP1 promotes formation of USP28 monomers and stabilizes MYC.

### Depletion of 53BP1 stimulates DNA replication and replication-dependent DNA damage

**Figure 6.**
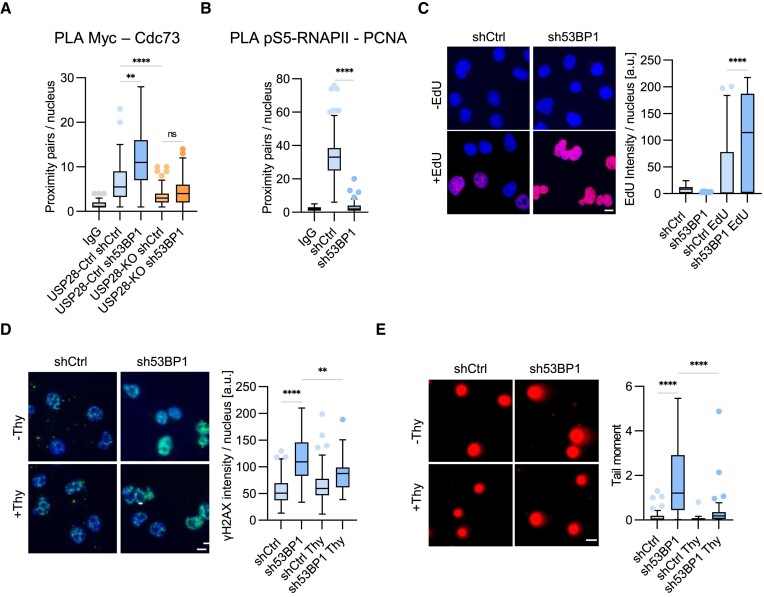
Knockdown of 53BP1 reduces TRCs and stimulates DNA synthesis. **(A)** PLA assays with antibodies against MYC and CDC73/IgG in HLF USP28-Ctrl/KO cells with shCtrl/sh53BP1. Quantification shows data points for one representative experiment (*n* = 2). At least 70 cells were quantified. The data were analyzed with Kruskal-Wallis test followed by Dunn's multiple comparison of selected pairs, **P < 0.01, ****P < 0.0001, ns *P* > 0.05. **(B)** PLA assays with antibodies against pS5-RNAPII and PCNA/IgG in HLF shCtrl/sh53BP1 cells. Quantification shows data points for one representative experiment (*n* = 3). At least 101 cells were quantified. The data were analyzed with two-tailed, Mann–Whitney test, ****P < 0.0001. **(C)** EdU incorporation assays in HLF shCtrl/sh53BP1 cells. Quantification shows data points for one representative experiment (*n* = 3). At least 69 cells were quantified. The data were analyzed with Kruskal-Wallis test followed by Dunn's multiple comparison of selected pair, ****P < 0.0001. Scale bar = 10 μm. **(D)** Immunofluorescence analysis with γH2AX antibodies in HLF shCtrl/sh53BP1 cells with or without thymidine treatment (2 mM, 2 hr). Quantification shows data points for one representative experiment (*n* = 2). At least 60 cells were quantified. The data were analyzed with Kruskal-Wallis test followed by Dunn's multiple comparison of selected pairs, **P < 0.01, ****P < 0.0001. Scale bar = 10 μm. **(E)** Neutral comet assays showing the DSBs in HLF shCtrl/sh53BP1 cells with or without thymidine treatment (2 mM, 2 h). Quantification shows data points for one representative experiment (*n* = 2). At least 34 cells were quantified. The data were analyzed with Kruskal–Wallis test followed by Dunn's multiple comparison of selected pairs, ****P < 0.0001. Scale bar = 10 μm.

Opposite to the effects of USP28 knockout, depletion of 53BP1 stimulated MYC interaction with PAF1c (Figure 6A, Supplementary Figure S6A). Importantly, this effect was abolished in USP28-KO cells expressing sh53BP1, demonstrating that activation of USP28 in 53BP1-deficient cells underlies enhanced MYC-PAF1c interaction. Knockdown of 53BP1 also stimulated PAF1c interaction with pS5-RNAPII (Supplementary Figure S6B), consistent with the idea that 53BP1 limits accumulation of PAF1c at active promoters. PLA assays with antibodies against pS5-RNAPII and PCNA showed a reduction in proximity pairs in sh53BP1 cells compared to shCtrl (Figure 6B), indicative of lower incidence of TRCs in the absence of 53BP1. EdU incorporation was increased in 53BP1-depleted cells relative to control (Figure 6C), mimicking the phenotype of USP28-M cells (Figure 2E). The enhanced EdU incorporation was reverted by the knockout of USP28 (Supplementary Figure S6C), in line with the model that ectopic DNA synthesis upon loss of 53BP1 is mediated by USP28.

Loss of 53BP1 activates BRCA1-dependent DNA repair by homologous recombination (HR) (66), which could stimulate EdU incorporation in 53BP1-deficient cells. To estimate the extent of HR in our assays, we treated cells with mirin, an inhibitor of the Mre11 exonuclease activity, required for HR (67). Mirin only slightly reduced the EdU incorporation in sh53BP1 cells (Supplementary Figure S6D), showing that HR does not significantly contribute to the enhanced DNA synthesis upon knockout of 53BP1.

Depletion of 53BP1 increased levels of γH2AX (Figure 6D), which was reverted by incubation with thymidine (Figure 6D), indicative of replication-dependent DNA damage. Consistently, neutral comet assays revealed elevated levels of DNA breakage in sh53BP1 cells compared to shCtrl cells (Figure 6E), which was rescued by addition of thymidine (Figure 6E). Importantly, deletion of USP28 alleviated the increase in γH2AX levels and in comet tail length, induced by knockdown of 53BP1 (Supplementary Figure S6E and F), arguing that activation of USP28 contributes to DNA breakage in 53BP1-deficient cells.

### Etoposide triggers a transient replicative response via 53BP1 and USP28

**Figure 7.**
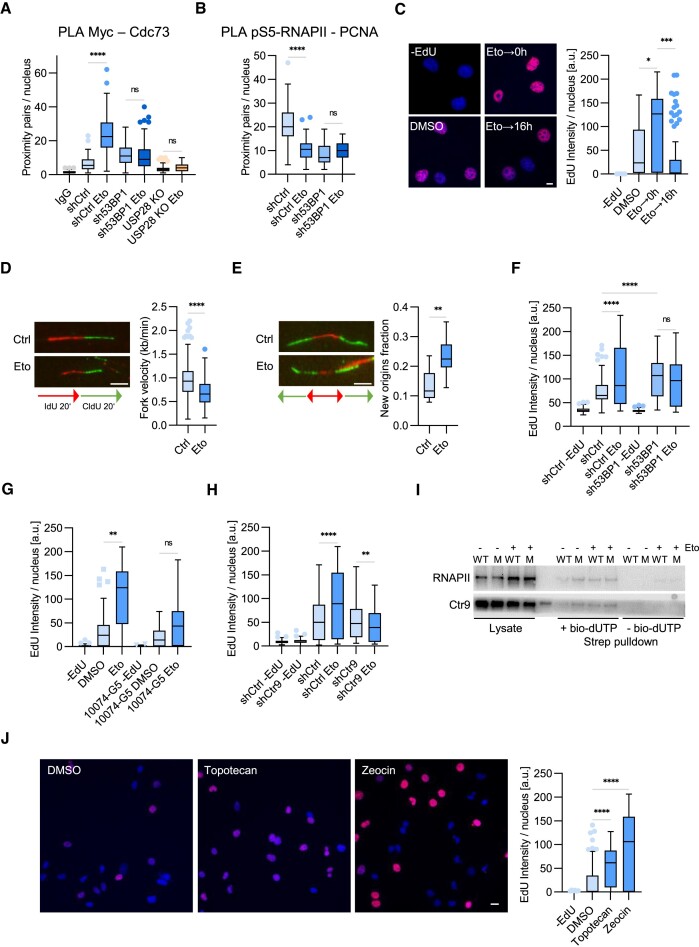
Etoposide triggers a transient replicative response via 53BP1 and USP28. **(A)** PLA assays with antibodies against MYC and CDC73/IgG in HLF shCtrl/sh53BP1 or USP28-KO cells with or without etoposide treatment (5 μM, 30 min). Quantification shows data points for one representative experiment (*n* = 2). At least 41 cells were quantified. The data were analyzed with Kruskal–Wallis test followed by Dunn's multiple comparison of selected pairs, ****P < 0.0001, ns *P* > 0.05. **(B)** PLA assays with antibodies against pS5-RNAPII and PCNA in HLF shCtrl/sh53BP1 cells with or without etoposide treatment (5 μM, 30 min). Quantification shows data points for one representative experiment (*n* = 3). At least 44 cells were quantified. The data were analyzed with Kruskal–Wallis test followed by Dunn's multiple comparison of selected pairs, ****P < 0.0001, ns *P* > 0.05. **(C)** EdU incorporation assays in HLF cells with DMSO or etoposide treatment (5 μM, 30 min) with or without release (16 h). Quantification shows data points for one representative experiment (*n* = 2). At least 69 cells were quantified. The data were analyzed with Kruskal–Wallis test followed by Dunn's multiple comparison of selected pairs, *P < 0.05, ***P < 0.001. Scale bar = 10 μm. **(D)** DNA fiber assays in HLF cells with or without etoposide treatment (5 μM, 30 min). Quantification shows data points for one representative experiment (*n* = 3). At least 263 fibers were quantified. The data were analyzed with two-tailed, Mann-Whitney test, ****P < 0.0001. Scale bar = 5 μm. **(E)** Quantification of new origins fraction from data shown in panel **(D)**. The data were analyzed with two-tailed, unpaired *t* test, **P < 0.01. Scale bar = 5 μm. **(F)** EdU incorporation assays in HLF shCtrl/sh53BP1 cells with or without etoposide treatment (5 μM, 30 min). Quantification shows data points for one representative experiment (*n* = 3). At least 124 cells were quantified. The data were analyzed with Kruskal-Wallis test followed by Dunn's multiple comparison of selected pairs, ****P < 0.0001, ns *P* > 0.05. **(G)** EdU incorporation assays in HLF cells with 10074-G5 (10 μM, 2 h) and etoposide (5 μM, 30 min) alone or combined treatment. Quantification shows data points for one representative experiment (*n* = 2). At least 43 cells were quantified. The data were analyzed with Kruskal-Wallis test followed by Dunn's multiple comparison of selected pairs, **P < 0.01, ns *P* > 0.05. **(H)** EdU incorporation assays in HLF shCtrl/shCTR9 cells with or without etoposide treatment (5 μM, 30 min). Quantification shows data points for one representative experiment (*n* = 2). At least 164 cells were quantified. The data were analyzed with Kruskal-Wallis test followed by Dunn's multiple comparison of selected pairs, **P < 0.01, ****P < 0.0001. **(I)** Immunoblotting analysis of protein levels of RNAPII and CTR9 on nascent chromatin captured from lysates of HLF USP28-WT or USP28-M cells with or without etoposide treatment (5 μM, 30 min). Image shows one representative experiment (*n* = 2). **(J)** EdU incorporation assays in HLF cells with or without topotecan or zeocin treatment (1 μM for topotecan and 100 μg/ml for zeocin, 30 min). At least 81 cells were quantified. The data were analyzed with Kruskal–Wallis test followed by Dunn's multiple comparison of selected pairs, ****P < 0.0001. Scale bar = 10 μm.

Treatment with etoposide stimulated recruitment of PAF1c to MYC in control cells with a negligible effect in sh53BP1 or USP28-KO cells (Figure 7A). Furthermore, etoposide decreased TRCs (the RNAPII–PCNA PLA pairs) in shCtrl cells but not in sh53BP1 cells (Figure 7B), suggesting that dissociation of 53BP1 from USP28 dimers promotes PAF1c recruitment and resolution of TRCs upon genotoxic stress. RNA synthesis was reduced in both etoposide-treated shCtrl and sh53BP1 cells (Supplementary Figure S7A), arguing that transcription does not account for differential effects on TRCs in the two cell lines.

Since the decrease in TRCs in USP28-M and sh53BP1 cells is accompanied by enhanced DNA synthesis, we assessed EdU incorporation at different timepoints after a short (30min) exposure to etoposide. DNA synthesis was reduced at 16h after release from the treatment (Figure 7C), likely reflecting the DNA damage-induced cell cycle arrest. In contrast, immediately after etoposide release, EdU incorporation was increased compared to unchallenged cells (Figure 7C). EdU incorporation was strongly affected by Mre11 inhibition at late timepoints, but not early after etoposide release (Supplementary Figure S7B), indicating that HR-mediated repair does not significantly contribute to etoposide-induced DNA synthesis.

DNA fiber assays showed that replication fork progression was slowed by etoposide (Figure 7D). However, the fraction of fibers, identified as new origins (49), strongly increased after treatment (Figure 7E), suggesting that etoposide- induced EdU incorporation is due to ectopic origin firing. Etoposide did not stimulate EdU incorporation in USP28-KO cells (Supplementary Figure S7C). Both sh53BP1 and USP28-M elevated basal EdU incorporation but diminished the effect of etoposide in HLF cells (Figure 7F, Supplementary Figure S7D). Deletion of 53BP1 in HeLa cells also abolished etoposide-induced DNA synthesis (Supplementary Figure S7E), suggesting that genotoxic stress can induce DNA replication in a 53BP1-dependent manner in different cellular contexts. ATM inhibition diminished the increase in EdU incorporation upon etoposide treatment in shCtrl cells but not in sh53BP1 cells (Supplementary Figure S7F), correlating with effects on USP28 dimers (Supplementary Figure S4E). Chemical inhibition of MYC-Max interaction with 10074-G5 (68) and depletion of CTR9 and CDC73 also reduced etoposide-induced DNA synthesis (Figure 7G and H; Supplementary Figure S7G), arguing that recruitment of PAF1c by MYC, at least in part, mediates this response. Nascent chromatin capture assays revealed an accumulation of RNAPII and CTR9 on nascent DNA upon etoposide treatment of cells expressing USP28-WT. This effect of etoposide was blunted in cells expressing USP28-M, indicating that ectopic DNA synthesis upon genotoxic stress involves formation of USP28 monomers (Figure 7I).

We then tested the impact of other genotoxins on DNA synthesis. Treatment of HLF cells with either topotecan or zeocin, which induce single and double strand breaks, respectively, stimulated EdU incorporation (Figure 7J), suggesting that transient stimulation of DNA replication is a common early response to genotoxic stress.

### Etoposide-induced DNA synthesis propagates DNA damage

**Figure 8.**
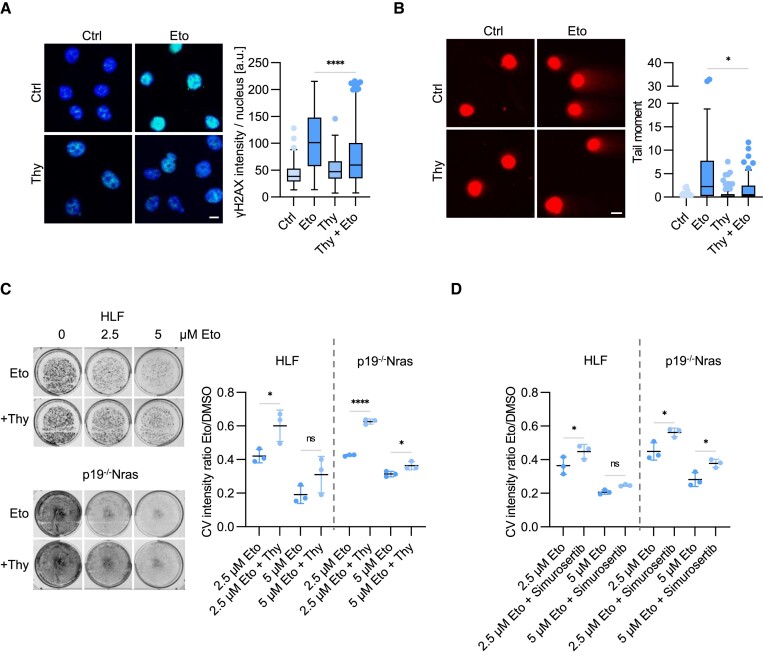
Etoposide-induced DNA synthesis propagates DNA damage. **(A)** Immunofluorescence analysis of γH2AX intensity in HLF cells with etoposide (5 μM, 30 min) and thymidine (2 mM, 2 h) alone or combined treatment. Quantification shows data points for one representative experiment (*n* = 2). At least 77 cells were quantified. The data were analyzed with Kruskal-Wallis test followed by Dunn's multiple comparison of selected pair, ****P < 0.0001. Scale bar = 10 μm. **(B)** Neutral comet assays showing the DSBs in HLF cells with etoposide (5 μM, 30 min) and thymidine (2 mM, 2 h) alone or combined treatment. Quantification shows data points for one representative experiment (*n* = 2). At least 78 cells were quantified. The data were analyzed with Kruskal-Wallis test followed by Dunn's multiple comparison of selected pair, *P < 0.05. Scale bar = 10 μm. **(C)** Crystal violet staining showing etoposide (2.5 or 5 μM, 30 min) treated HLF and p19^−/−^Nras cells with or without thymidine (2 mM, 1 h prior etoposide treatment). Right panels show the mean of three independent biological replicates (*n* = 3). The data were analyzed with ordinary one-way ANOVA followed by Šídák's multiple comparison of selected pairs, *P < 0.05, ****P < 0.0001, ns *P* > 0.05. Error bars denote S.D. **(D)** Crystal violet staining quantifications of etoposide (2.5 or 5 μM, 30 min) treated HLF and p19^−/−^Nras cells with or without CDC7 inhibitor Simurosertib (2 μM, 1 h prior etoposide treatment) showing the mean of three independent biological replicates (*n* = 3). The data were analyzed with ordinary one-way ANOVA followed by Šídák's multiple comparison of selected pairs, *P < 0.05, ns *P* > 0.05. Error bars denote S.D.

Ectopic DNA replication upon etoposide treatment may promote DNA breakage, as observed in cells expressing sh53BP1 and USP28-M (Figures 3 and 6). Supporting this idea, PLA assays showed a strong increase in association of γH2AX with nascent DNA following EdU labelling after a brief exposure to etoposide (Supplementary Figure S8A). To directly test the impact of DNA replication on etoposide-induced DNA breakage, we treated cells with etoposide alone or in combination with thymidine. Immunofluorescence analysis showed that co-treatment with thymidine diminished etoposide-induced increase in γH2AX levels (Figure 8A). Neutral comet assays revealed reduced DNA breakage in cells co-treated with thymidine compared to etoposide treatment alone (Figure 8B), arguing that aberrant stimulation of DNA replication by etoposide propagates DNA damage. Thymidine also diminished etoposide-induced cytotoxicity and promoted long term survival of HLF, p19-/-Nras and HeLa cells (Figure 8C; Supplementary Figure S8B). A similar effect was observed upon inhibition of the CDC7 kinase, which is specifically required for initiation of DNA replication (69) (Figure 8D). In contrast, inhibition of PARP1 to accelerate the progression of DNA replication forks (58), increased etoposide-mediated cytotoxicity in both HLF, p19-/-Nras and HeLa cells (Supplementary Figure S8C). We conclude that the early replicative response to genotoxic stress exacerbates DNA breakage and impairs cell viability.

## Discussion

Here, we provide evidence that dimerization of USP28 attenuates deubiquitination of MYC and limits recruitment of the elongation factor PAF1c. USP28 dimers are disassembled upon genotoxic stress, leading to ectopic PAF1c recruitment, resolution of TRCs and transient stimulation of DNA synthesis.

USP28 interacts with MYC and other oncogenic transcription factors, such as Jun and Notch, via the common ubiquitin ligase SCF(FBW7) that recognizes specific phosphodegrons (70). USP28 can also bind substrates directly or via other adaptor proteins (62,71). While FBW7 primarily assembles K48-linked ubiquitin chains, other MYC ubiquitin ligases can conjugate different types of chains, including K63 and K11 (36,46,70,72–75). We find that monomeric USP28 has an enhanced ability to deubiquitinate MYC and is selectively active towards K11-linked chains, suggesting that such chains contribute to constitutive MYC turnover during unperturbed cell cycle. K11 chains are predominantly assembled by the anaphase-promoting complex Apc/c, but also by other ligases that target MYC including Huwe1, RNF4, RNF8 and beta-TrCP (46,72,74,76–78). USP28 monomers may antagonize ubiquitination of MYC by these enzymes, analogously to the mechanism described for Apc/c-mediated turnover of Claspin (79).

Ubiquitination is essential for MYC transcriptional function (36,37,39). In particular, ubiquitination of MYC promotes histone acetylation, recruitment of elongation factors pTEFb and PAF1c, and the transfer of PAF1c from MYC onto RNAPII (27). PAF1c is a multivalent complex controlling transcriptional pausing, processive elongation, RNA maturation and nuclear export (80,81). PAF1c also facilitates resolution of TRCs and promotes homologous recombination-dependent DNA repair by stimulating ubiquitination of histone H2B (26,28,82,83). In line, recruitment of PAF1c to MYC was recently shown to be dependent on the Huwe1 ubiquitin ligase and suggested to mediate DNA repair at transcription start sites (26). However, other studies found that PAF1c can lead to the accumulation of R-loops and promote genomic instability under replicative stress (30,31). We show that ectopic stabilization of MYC by monomeric USP28 stimulates recruitment of PAF1c and resolution of TRCs to drive ectopic DNA synthesis. These findings support and extend previous studies on MYC-driven DNA replication and recent reports that TRCs can limit ectopic DNA replication and promote DNA repair under stress (21,23,84,85).

USP28 is thought to exist predominantly as dimers (42), raising the question of what induces formation of USP28 monomers. We show that USP28 dimerization is stimulated by 53BP1—the major binding partner of USP28 and a key mediator protein in cellular response to DNA damage and replicative stress (41,86–89). 53BP1 selectively interacts with USP28 dimers and depletion of 53BP1 favors USP28 monomers, suggesting that 53BP1 stabilizes the dimeric conformation of USP28. Consistently, loss of 53BP1 mimics expression of monomeric USP28 with increased PAF1 recruitment and accumulation of replication-dependent DNA damage.

The 53BP1–USP28 interaction is diminished upon genotoxic stress in an ATM-dependent manner, leading to formation of USP28 monomers and stabilization of MYC. This can provide a simple mechanism for the activation of USP28 upon DNA damage and other stresses, such as prolonged mitosis and disruption of centrosomes (40,41,64,90–92). The molecular underpinnings of the disassembly of 53BP1–USP28 complexes upon genotoxic stress remain to be investigated but can involve proteasomal degradation of 53BP1 (93) or its recruitment to modified histones at DNA damage sites (3), which could sterically interfere with USP28 binding.

In our model, dimerization of USP28 limits unscheduled DNA replication at transcriptionally active loci by preventing ectopic recruitment of PAF1 to MYC. We propose that during an unperturbed cell cycle, DDR signaling due to endogenous DNA lesions or fork stalling can disrupt USP28 dimers to transiently activate USP28 for localized origin firing. By contrast, constitutive activation of USP28 by mutation of the dimer interface or upon loss of 53BP1 leads to chronic stimulation of DNA synthesis accompanied by the accumulation of DNA damage.

Our data indicate that USP28 monomers primarily form in response to genotoxic stress leading to PAF1 recruitment and stimulating DNA replication. Under these conditions the progression of replication forks is slowed and the net stimulation of DNA replication is most likely due to firing of dormant origins, which are thought to localize in the vicinity of transcription start sites (94,95). Previous studies suggested that firing of dormant origins during recovery from stress provides a mechanism to replicate DNA regions trapped between broken replication forks (96,97). In line with this model, our data indicate that ATM-dependent activation of USP28 stimulates origin firing early after genotoxic stress. However, replication inhibitors diminish genotoxin-induced DNA damage and cytotoxicity, arguing that origin firing upon genotoxic stress is associated with increased DNA breakage. Apparently, ectopic origin firing enforces S phase progression, even at the cost of replicating damaged DNA. If the initial damage is low, this may favor HR-mediated DNA repair in S and G2 phases, over error-prone non-homologous end joining, which is predominant in G1. If the repair fails, the exacerbated DNA damage can effectively drive cells into apoptosis or senescence to safeguard against accumulation of oncogenic lesions. Alternatively, genotoxin-induced origin firing may reflect an irrational pathological reaction to genotoxic stress when cells fail to arrest in the G1 phase. In either case, our observations warrant further analysis of genotoxin-induced DNA replication and suggest that it can be exploited for the development of targeted combinatorial therapies.

## Supplementary Material

gkae004_Supplemental_File

## Data Availability

All data needed to evaluate the conclusions in the paper are present in the paper and/or the Supplementary Materials. The RNA-seq data are deposited at the NCBI GEO portal under accession number GSE213892. The proteomic data are deposited at the PRIDE database under PXD037263.

## Notes

Present address: Wenshan Xu, No. 1000, Hanghe Road, Pudong New Area, Shanghai 201317, China.

Present address: Ravi Babu Kollampally, Institute of Molecular Infection Biology, University of Würzburg, Josef-Schneider-Straße 2, 97080 Würzburg, Germany.
