# Supplementary material for: The dimeric deubiquitinase USP28 integrates 53BP1 and MYC functions to limit DNA damage
Source: Nucleic Acids Res. 2024 Jan 16;52(6):3011–30. doi: 10.1093/nar/gkae004 (PMC11024517; doi:10.1093/nar/gkae004)
Supplement: gkae004_Supplemental_File [file gkae004_supplemental_file.pdf]

The dimeric deubiquitinase USP28 integrates 53BP1 and MYC functions to limit DNA damage

Jin et al.

Supplementary Information

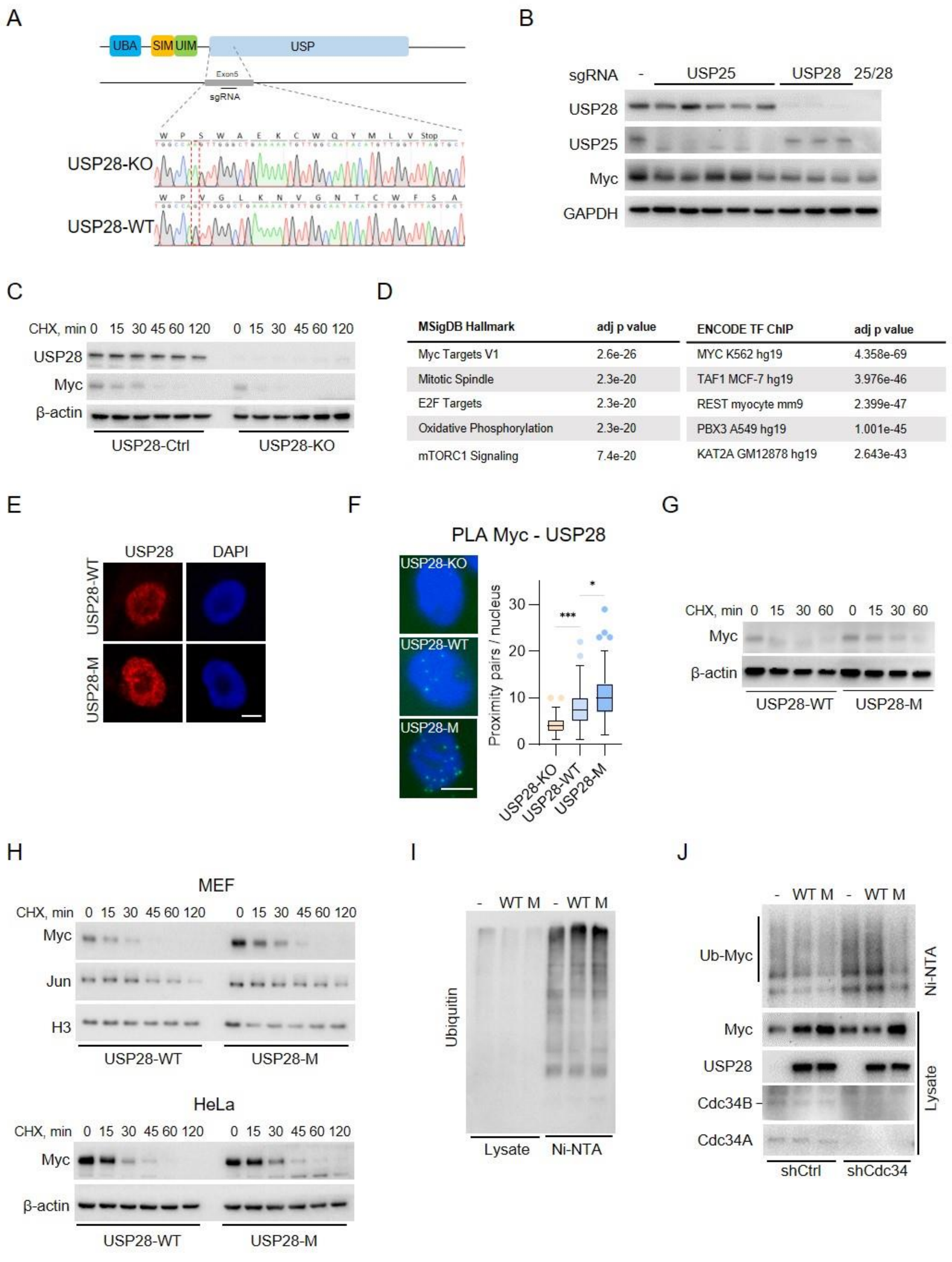
A
UBA
SIM
UIM
USP
Exon5
sgRNA
USP28-KO
W P S W A E K C W Q Y M L V Stop
USP28-WT
W P V G L K N V G N T C W F S A
B
sgRNA - USP25 USP28 25/28
USP28
USP25
Myc
GAPDH
C
CHX, min 0 15 30 45 60 120 0 15 30 45 60 120
USP28
Myc
β-actin
USP28-Ctrl
USP28-KO
D
MSigDB Hallmark
adj p value
Myc Targets V1
2.6e-26
Mitotic Spindle
2.3e-20
E2F Targets
2.3e-20
Oxidative Phosphorylation
2.3e-20
mTORC1 Signaling
7.4e-20
ENCODE TF ChIP
adj p value
MYC K562 hg19
4.358e-69
TAF1 MCF-7 hg19
3.976e-46
REST myocyte mm9
2.399e-47
PBX3 A549 hg19
1.001e-45
KAT2A GM12878 hg19
2.643e-43
E
USP28
DAPI
USP28-WT
USP28-M
F
PLA Myc - USP28
USP28-KO
USP28-WT
USP28-M
Proximity pairs / nucleus
0
10
20
30
***
*
USP28-KO
USP28-WT
USP28-M
G
CHX, min 0 15 30 60 0 15 30 60
Myc
β-actin
USP28-WT
USP28-M
H
MEF
CHX, min 0 15 30 45 60 120 0 15 30 45 60 120
Myc
Jun
H3
USP28-WT
USP28-M
HeLa
CHX, min 0 15 30 45 60 120 0 15 30 45 60 120
Myc
β-actin
USP28-WT
USP28-M
I
- WT M - WT M
Ubiquitin
Lysate
Ni-NTA
J
- WT M - WT M
Ub-Myc
Ni-NTA
Myc
USP28
Cdc34B
Cdc34A
Lysate
shCtrl
shCdc34

Fig. S1, related to Fig. 1.

(A) Schematic of CRISPR-based deletion of USP28 used in HLF cells and the Sanger sequencing data of a control and a knockout clone.

(B) Immunoblot analysis of protein levels of USP25, USP28 and MYC in different clones of HLF cells with sgRNA against USP25 and/or USP28. Note that a faint band is detected by the USP28 antibody in USP28-KO clones, as was also the case for the Cre-mediated knockout of USP28 in murine cells (45). This is most likely due to cross reactivity of the antibody with the highly homologous USP25 protein, as the band is absent in the USP28/USP25 double knockout cells.

(C) Immunoblotting analysis of MYC protein level in HLF USP28-Ctrl/KO cells, treated with cycloheximide (100 µg/ml) for the indicated time points. Image shows one representative biological experiment (n=3).

(D) Top five gene sets for the indicated databases, enriched within the USP28-deregulated genes based on the analysis of RNA-seq data in HLF USP28-KO and USP28-Ctrl cells. Analysis by the Enrichr portal (98).

(E) Immunofluorescence analysis documenting nuclear localization of wildtype (WT) and monomeric (M) USP28 in HLF cells. Scale bar = 10 µm.

(F) PLA assays with antibodies against MYC and USP28 in HLF USP28-KO cells expressing USP28-WT/M or a control vector. Quantification shows data points for one representative experiment (n=2). At least 30 cells were quantified. The data were analyzed with Kruskal-Wallis test followed by Dunn's multiple comparison of selected pairs, *P < 0.05, ***P < 0.001. Scale bar = 10 µm.

(G) Immunoblotting analysis of $p19^{-/-}$Nras cells, expressing USP28-WT or USP28-M, treated with cycloheximide (100 µg/ml) for the indicated time points. Image shows one representative experiment (n=2).

(H) Immunoblotting analysis of USP28-KO MEFs and HeLa cells, expressing USP28-WT or USP28-M, treated with cycloheximide (100 µg/ml) for the indicated time points. Image shows one of two representative experiments (n=2).

(I) Ubiquitin pulldown assays with HeLa USP28-KO cells expressing MYC, WT His-Ub and USP28-WT/M, immunoblotting with antibodies against total ubiquitin. Image shows one representative experiment (n=2).

(J) Ubiquitin pulldown assays with HeLa USP28-KO cells expressing MYC, K11-only His-Ub and USP28-WT/M, co-transfected with shCtrl or shCDC34A/B. Image shows one representative experiment (n=3).

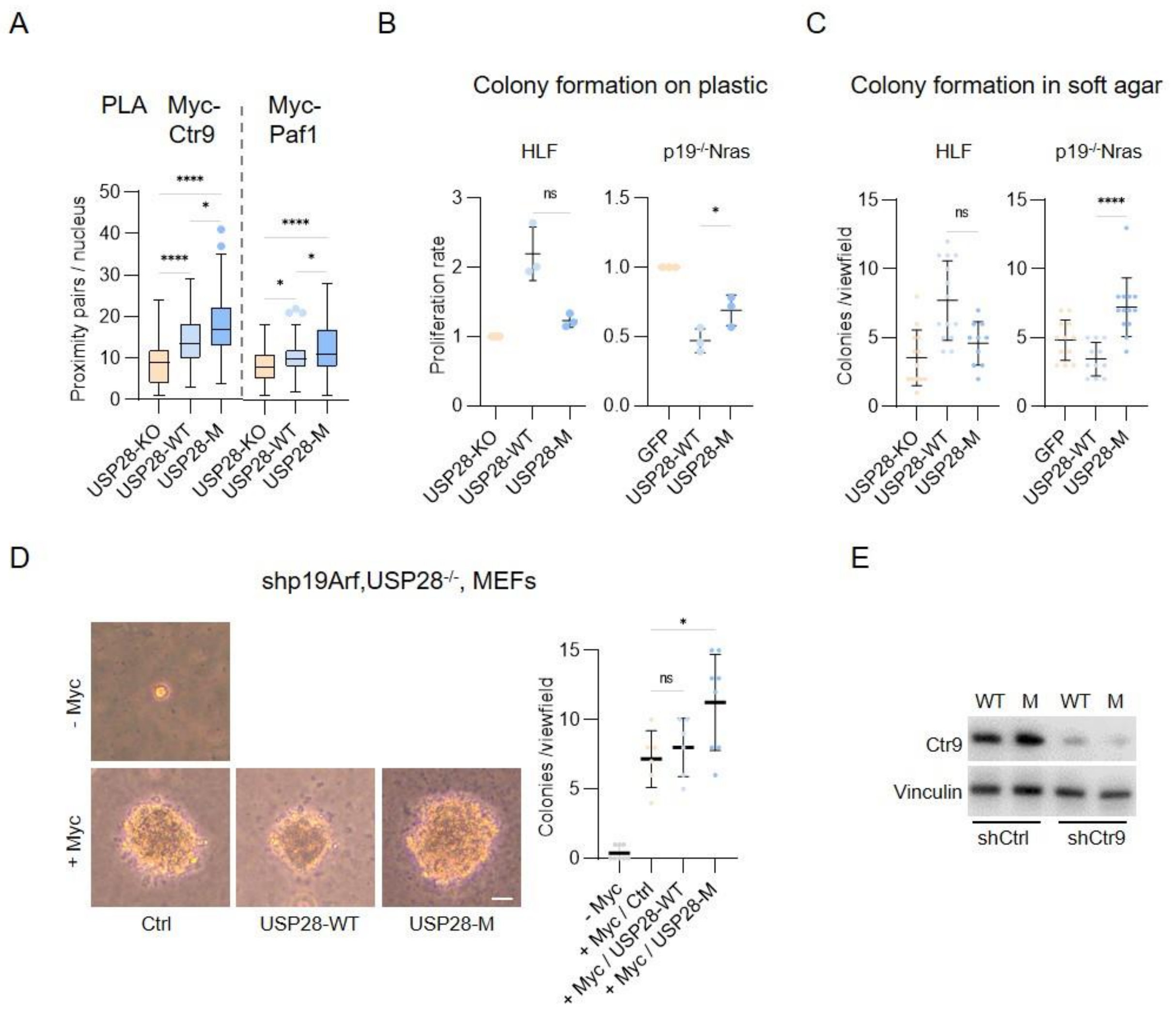
A
B
C
PLA Myc-Ctr9
Myc-Paf1
Proximity pairs / nucleus
USP28-KO
USP28-WT
USP28-M
Colony formation on plastic
HLF
p19-/-Nras
Proliferation rate
GFP
Colony formation in soft agar
Colonies /viewfield
D
shp19Arf,USP28-/-, MEFs
- Myc
+ Myc
Ctrl
USP28-WT
USP28-M
- Myc
+ Myc / Ctrl
+ Myc / USP28-WT
+ Myc / USP28-M
E
WT
M
Ctr9
Vinculin
shCtrl
shCtr9

Fig. S2, related to Fig. 2.

(A) PLA assays with antibodies against MYC and CTR9 or PAF1 in HLF USP28-KO cells expressing USP28-WT/M or a control vector. Quantification shows data points for one representative experiment (n=2). At least 65 cells were quantified. The data were analyzed with Kruskal-Wallis test followed by Dunn's multiple comparison of selected pairs, $^*P < 0.05$, $^{****}P < 0.0001$.

(B) Crystal violet staining showing proliferation rate of HLF (left panel) or $p19^{-/-}$Nras (right panel) cells expressing USP28-WT/M. Panels show the mean of three independent biological replicates (n=3). The data were analyzed with Kruskal-Wallis test followed by Dunn's multiple comparison of selected pair (HLF) or with ordinary one-way ANOVA followed by Šídák's multiple comparison of selected pair ($p19^{-/-}$Nras), $^*P < 0.05$, ns $P > 0.05$. Error bars denote S.D.

(C) Colony formation assay in soft agar for HLF (left panel) or $p19^{-/-}$Nras (right panel) cells with USP28-WT/M expression. Quantification shows data points for one representative experiment (n=2). At least 10 (HLF) or 11 ($p19^{-/-}$Nras) view fields were quantified. The data were analyzed with Kruskal-Wallis test followed by Dunn's multiple comparison of selected pair, $^{****}P < 0.0001$, ns $P > 0.05$. Error bars denote S.D.

(D) Colony formation assay in soft agar for USP28-KO MEFs, expressing an shRNA against p19Arf and vectors encoding USP28-WT, USP28-M or a control vector. Quantification shows data points for one representative experiment (n=4). At least 6 view fields were quantified. The data were analyzed with ordinary one-way ANOVA followed by Tukey's multiple comparison test of selected pairs, $^*P < 0.05$, ns $P > 0.05$. Error bars denote S.D. Scale bar = 10 μm.

(E) Immunoblotting analysis documenting CTR9 depletion in HLF USP28-WT or USP28-M cells with shCtrl/shCTR9.

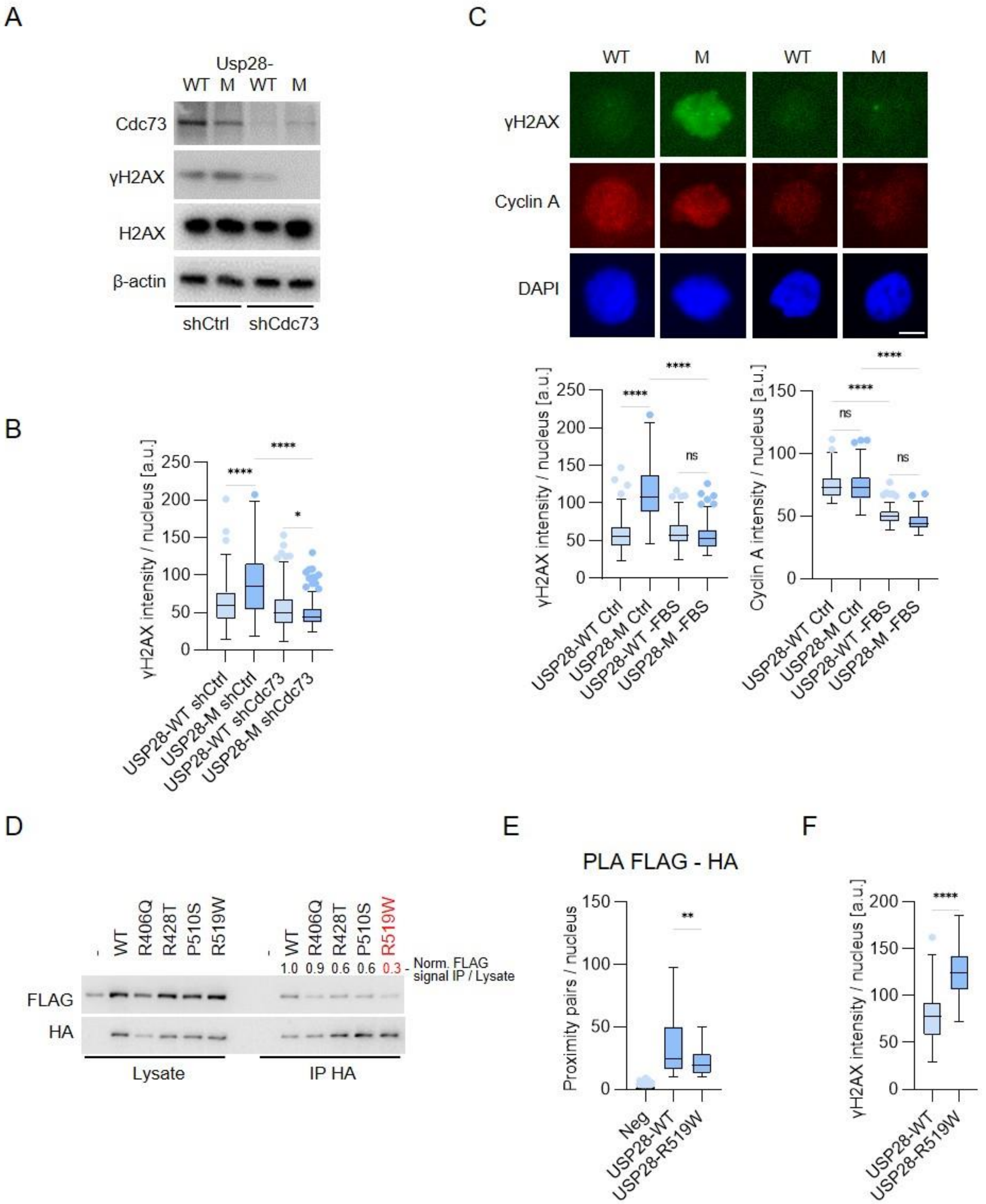
A
Usp28-
WT M WT M
Cdc73
γH2AX
H2AX
β-actin
shCtrl shCdc73
B
γH2AX intensity / nucleus [a.u.]
250
200
150
100
50
0
****
****
*
USP28-WT shCtrl
USP28-M shCtrl
USP28-WT shCdc73
USP28-M shCdc73
C
WT M WT M
γH2AX
Cyclin A
DAPI
γH2AX intensity / nucleus [a.u.]
****
****
ns
USP28-WT Ctrl
USP28-M Ctrl
USP28-WT -FBS
USP28-M -FBS
Cyclin A intensity / nucleus [a.u.]
****
****
ns
ns
D
- WT R406Q R428T P510S R519W
- WT R406Q R428T P510S R519W
1.0 0.9 0.6 0.6 0.3
Norm. FLAG signal IP / Lysate
FLAG
HA
Lysate
IP HA
E
PLA FLAG - HA
Proximity pairs / nucleus
**
Neg
USP28-WT
USP28-R519W
F
γH2AX intensity / nucleus [a.u.]
****
USP28-WT
USP28-R519W

Fig. S3, related to Fig. 3.

(A) Immunoblotting analysis documenting γH2AX expression in HLF USP28-WT or USP28-M cells, expressing shCtrl/shCDC73. Image shows one representative experiment (n=3).

(B) Immunofluorescence analysis documenting γH2AX intensity in HLF USP28-WT or USP28-M cells, expressing shCtrl/shCDC73. Quantification shows data points for one representative experiment (n=3). At least 104 cells were quantified. The data were analyzed with Kruskal-Wallis test followed by Dunn's multiple comparison of selected pairs, *P < 0.05, ****P < 0.0001.

(C) Immunofluorescence analysis documenting γH2AX and Cyclin A intensity in HLF USP28-WT or USP28-M cells with or without serum deprivation. Quantification shows data points for one representative experiment (n=2). At least 93 cells were quantified. The data were analyzed with Kruskal-Wallis test followed by Dunn's multiple comparison of selected pairs, ****P < 0.0001, ns P > 0.05. Scale bar = 10 μm.

(D) Immunoprecipitation analysis with the HA antibodies in HeLa USP28-KO cells, expressing FLAG-tagged USP28-WT and HA-tagged wildtype or mutant USP28. Numbers show the ratio of FLAG grayscale intensity between IP and Lysate normalized to the control group.

(E) PLA assays with antibodies against FLAG and HA or IgG in HeLa USP28-KO cells, expressing FLAG-tagged USP28-WT and HA-tagged USP28-WT/R519W. Quantification shows data points for one representative experiment (n=2). At least 80 cells were quantified. The data were analyzed with two-tailed, Mann-Whitney test, **P < 0.01.

(F) Immunofluorescence analysis documenting γH2AX intensity in HeLa USP28-KO cells, expressing Flag-tagged USP28-WT and HA-tagged USP28-WT/R519W. Quantification shows data points for one representative experiment (n=2). At least 119 cells were quantified. The data were analyzed with two-tailed, Mann-Whitney test, ****P < 0.0001.

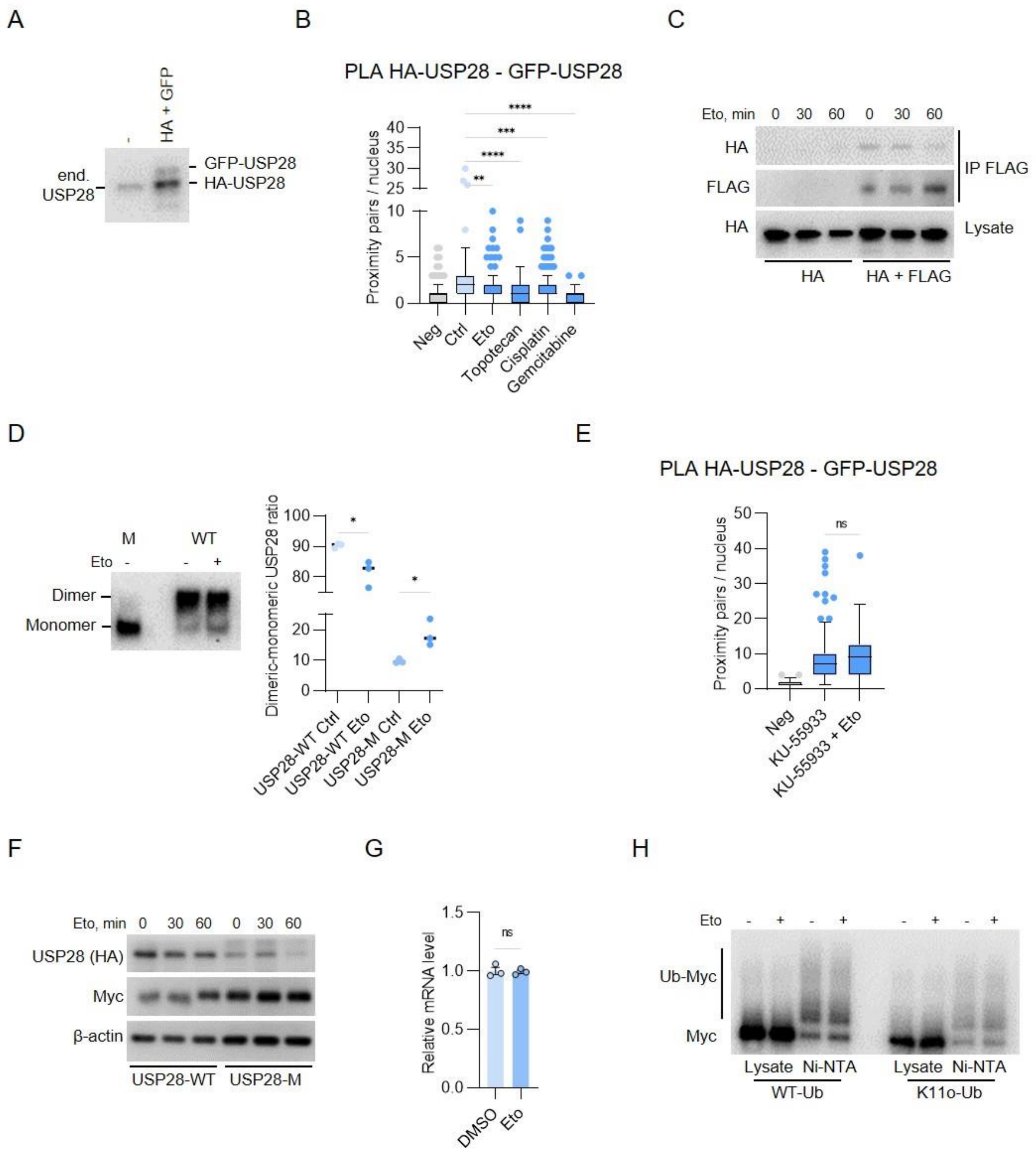
A
B
C
D
E
F
G
H
HA + GFP
end. USP28
GFP-USP28
HA-USP28
PLA HA-USP28 - GFP-USP28
Proximity pairs / nucleus
Neg
Ctrl
Eto
Topotecan
Cisplatin
Gemcitabine
Eto, min
0 30 60 0 30 60
HA
FLAG
IP FLAG
Lysate
HA + FLAG
M
WT
Dimer
Monomer
Dimeric-monomeric USP28 ratio
USP28-WT Ctrl
USP28-WT Eto
USP28-M Ctrl
USP28-M Eto
ns
KU-55933
KU-55933 + Eto
USP28 (HA)
Myc
β-actin
USP28-WT
USP28-M
Relative mRNA level
DMSO
Ub-Myc
Lysate
Ni-NTA
WT-Ub
K11o-Ub

Fig. S4, related to Fig. 4.

(A) Immunoblotting documenting expression of GFP- and HA-tagged USP28 proteins in p19$^{-/-}$Nras cells and endogenous USP28 in parental p19$^{-/-}$Nras cells.

(B) PLA assays with antibodies against GFP and HA-tag in p19$^{-/-}$Nras cells, expressing GFP- and HA-tagged USP28 with or without etoposide (5 μM, 30 min), topotecan (1 μM, 30 min), cisplatin (10 μM, 30 min), gemcitabine (10 μM, 30 min) treatment. At least 94 cells were quantified. The data were analyzed with Kruskal-Wallis test followed by Dunn's multiple comparison of selected pairs, **$P < 0.01$, ***$P < 0.001$, ****$P < 0.0001$.

(C) Immunoprecipitation analysis with FLAG antibodies from HeLa cells, transfected with HA- and FLAG-tagged USP28 before and after etoposide treatment (5 μM) for indicated time point. Image shows one representative experiment (n=2).

(D) Immunoblotting with Native PAGE documenting expression of USP28-M and the transition between USP28-WT and USP28-M after etoposide (5 μM, 30 min) treatment in HeLa USP28-KO cells transfected with USP28-WT/M. Right panel shows the mean of three independent biological replicates (n=3). The data were analyzed with ordinary one-way ANOVA test followed by Tukey's multiple comparison of selected pairs, *$P < 0.05$.

(E) PLA assays with antibodies against GFP and HA-tag in p19$^{-/-}$Nras shCtrl cells, expressing GFP- and HA-tagged USP28 with KU-55933 (2 μM, 2 hr) alone or combined with etoposide (5 μM, 30 min) treatment. At least 77 cells were quantified. The data were analyzed with two-tailed, Mann-Whitney test, ns $P > 0.05$.

(F) Immunoblotting documenting MYC protein levels in p19$^{-/-}$Nras cells, expressing USP28-WT/M, treated with etoposide (5 μM) for the indicated time points. Image shows one representative experiment (n=2).

(G) qPCR showing the mRNA level of MYC in HLF USP28-Ctrl cells with or without etoposide treatment (5 μM, 30 min). Quantification shows data points for one representative experiment (n=2). The data were analyzed from three technical replicates with two-tailed, unpaired t test, ns $P > 0.05$.

(H) His-Ub pulldown assay in HeLa USP28-KO cells, transfected with MYC and WT-Ub or K11-only His-Ub before and after etoposide (5 μM, 30 min) treatment. Image shows one representative experiment (n=2).

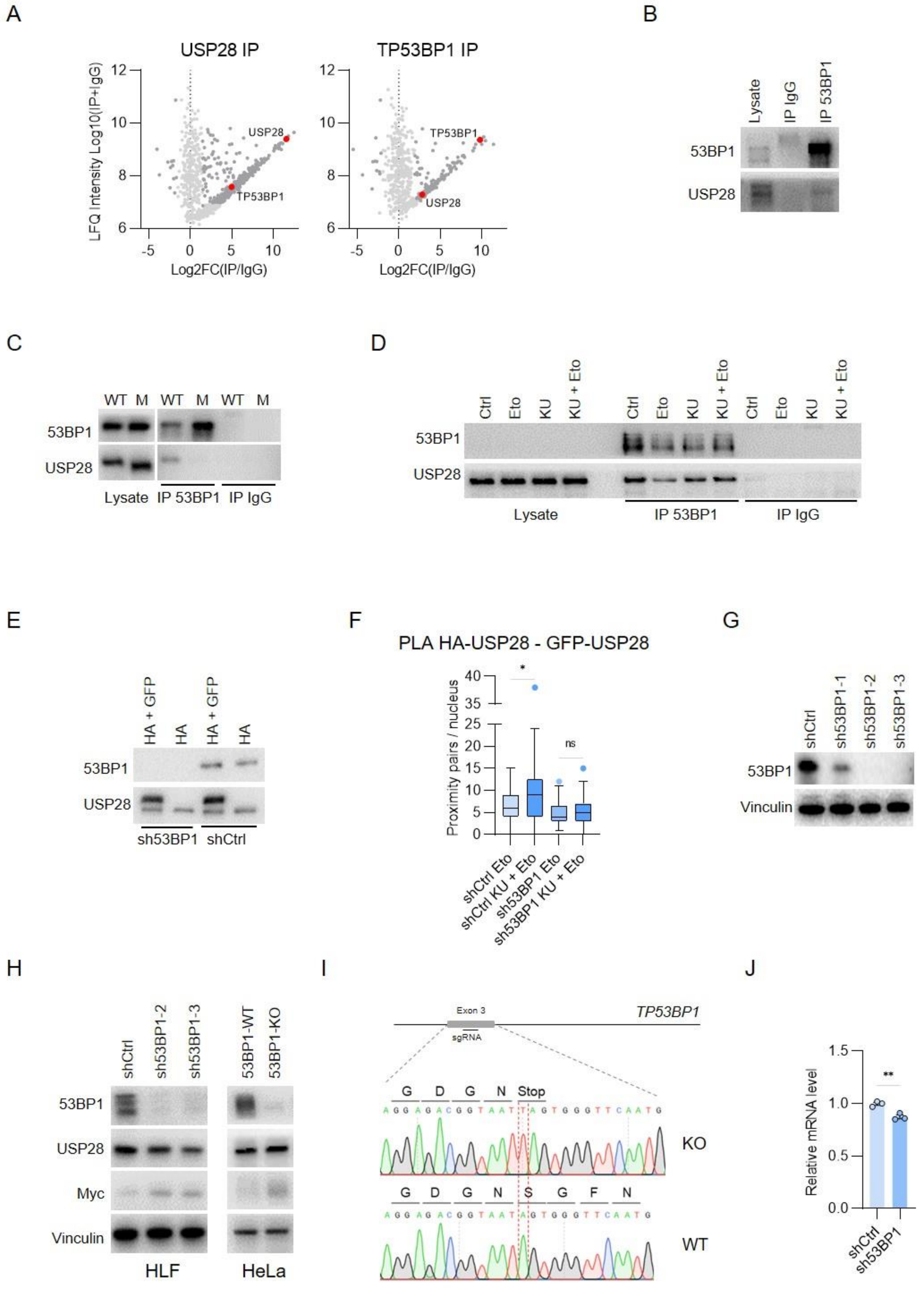
A
USP28 IP
TP53BP1 IP
LFQ Intensity Log10(IP+IgG)
Log2FC(IP/IgG)
USP28
TP53BP1
B
Lysate
IP IgG
IP 53BP1
53BP1
USP28
C
WT
M
Lysate
IP 53BP1
IP IgG
D
Ctrl
Eto
KU
KU + Eto
E
HA + GFP
HA
sh53BP1
shCtrl
F
PLA HA-USP28 - GFP-USP28
Proximity pairs / nucleus
ns
shCtrl Eto
shCtrl KU + Eto
sh53BP1 Eto
sh53BP1 KU + Eto
G
shCtrl
sh53BP1-1
sh53BP1-2
sh53BP1-3
Vinculin
H
53BP1-WT
53BP1-KO
Myc
HLF
HeLa
I
Exon 3
sgRNA
TP53BP1
G D G N Stop
G D G N S G F N
AGGAGACGGTAATTAGTGGGTTCAATG
AGGAGACGGTAATAGTGGGTTCAATG
KO
WT
J
Relative mRNA level
shCtrl
sh53BP1

Fig. S5, related to Fig. 5.

(A) LC-MS/MS analysis of immunoprecipitations with 53BP1 and USP28 antibodies from HeLa cells, showing reciprocal identification of USP28 and 53BP1. Dark grey dots denote significant interactors.

(B) Immunoprecipitation analysis with antibodies against 53BP1 or control IgG from HLF cells. Image shows one representative experiment (n=3).

(C) Immunoprecipitation analysis with antibodies against 53BP1 or control IgG from $p19^{-/-}$Nras cells expressing USP28-WT and USP28-M. Image shows one representative experiment (n=2).

(D) Immunoprecipitation analysis with antibodies against 53BP1 or control IgG from HLF cells with etoposide (5 μM, 30 min) and KU-55933 (2 μM, 2 hr) alone or combined treatment. Image shows one representative experiment (n=4).

(E) Immunoblotting documenting depletion of 53BP1 in $p19^{-/-}$Nras cells with HA- and/or GFP-tagged USP28.

(F) PLA assays with antibodies against GFP and HA-tag in $p19^{-/-}$Nras shCtrl/sh53BP1 cells, expressing GFP- and HA-tagged USP28, treated with etoposide alone (5 μM, 30 min) or in combination with KU-55933 (2 μM, 2 hr) treatment. The data for shCtrl cells with combined etoposide and KU-59933 treatment was the same as in Fig. S4E. At least 68 cells were quantified. The data were analyzed with Kruskal-Wallis test followed by Dunn's multiple comparison of selected pairs, $*P < 0.05$, ns $P > 0.05$.

(G) Immunoblots documenting depletion of 53BP1 in HLF cells.

(H) Immunoblots documenting USP28 and MYC protein levels in HLF-sh53BP1 and HeLa 53BP1KO cells.

(I) Schematic of CRISPR-based deletion of 53BP1 in HeLa cells and the resulting Sanger sequencing of the HeLa 53BP1-KO clone and a control clone.

(J) qPCR showing the mRNA level of MYC in HLF cells, expressing shCtrl or sh53BP1. Quantification shows data points for one representative experiment (n=2). The data were analyzed from three technical replicates with two-tailed, unpaired t test, $**P < 0.01$.

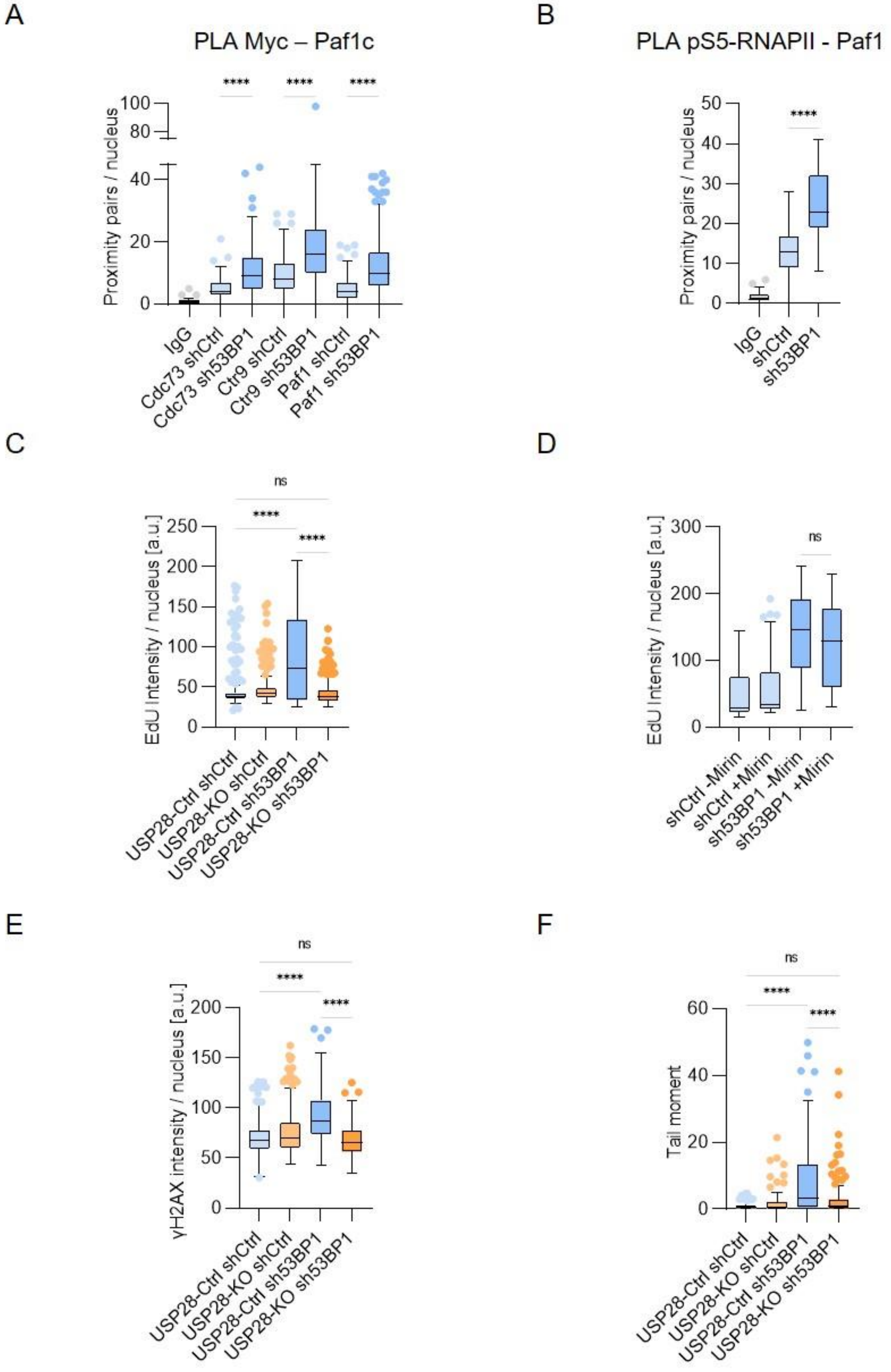

A
PLA Myc – Paf1c
Proximity pairs / nucleus
IgG
Cdc73 shCtrl
Cdc73 sh53BP1
Ctr9 shCtrl
Ctr9 sh53BP1
Paf1 shCtrl
Paf1 sh53BP1
B
PLA pS5-RNAPII - Paf1
Proximity pairs / nucleus
IgG
shCtrl
sh53BP1
C
EdU Intensity / nucleus [a.u.]
USP28-Ctrl shCtrl
USP28-KO shCtrl
USP28-Ctrl sh53BP1
USP28-KO sh53BP1
D
EdU Intensity / nucleus [a.u.]
shCtrl -Mirin
shCtrl +Mirin
sh53BP1 -Mirin
sh53BP1 +Mirin
E
γH2AX intensity / nucleus [a.u.]
USP28-Ctrl shCtrl
USP28-KO shCtrl
USP28-Ctrl sh53BP1
USP28-KO sh53BP1
F
Tail moment
USP28-Ctrl shCtrl
USP28-KO shCtrl
USP28-Ctrl sh53BP1
USP28-KO sh53BP1

Fig. S6, related to Fig. 6.

(A) PLA assays with antibodies against MYC and PAF1c subunits (CDC73/CTR9/PAF1) or IgG in HLF cells with shCtrl/sh53BP1. Quantification shows data points for one representative experiment (n=2). At least 130 cells were quantified. The data were analyzed with Kruskal-Wallis test followed by Dunn's multiple comparison of selected pairs, ****P < 0.0001.

(B) PLA assays with antibodies against pS5-RNAPII and PAF1/IgG in HLF shCtrl/sh53BP1 cells. Quantification shows data points for one representative experiment (n=2). At least 55 cells were quantified. The data were analyzed with two-tailed, unpaired t test, ****P < 0.0001.

(C) EdU incorporation assays in HLF USP28-Ctrl/KO cells with shCtrl/sh53BP1. Quantification shows data points for one representative experiment (n=2). At least 204 cells were quantified. The data were analyzed with Kruskal-Wallis test followed by Dunn's multiple comparison of selected pairs, ****P < 0.0001, ns P > 0.05.

(D) EdU incorporation assays in HLF shCtrl/sh53BP1 cells with or without Mirin treatment (25 mM, 2 hr). At least 52 cells were quantified. The data were analyzed with Kruskal-Wallis test followed by Dunn's multiple comparison of selected pair, ns P > 0.05.

(E) Immunofluorescence analysis with γH2AX antibodies in HLF USP28-Ctrl/KO cells with shCtrl/sh53BP1. Quantification shows data points for one representative experiment (n=2). At least 204 cells were quantified. The data were analyzed with Kruskal-Wallis test followed by Dunn's multiple comparison of selected pairs, ****P < 0.0001, ns P > 0.05.

(F) Neutral comet assays showing the DSBs in HLF USP28-Ctrl/KO cells with shCtrl/sh53BP1. Quantification shows data points for one representative experiment (n=2). At least 82 cells were quantified. The data were analyzed with Kruskal-Wallis test followed by Dunn's multiple comparison of selected pairs, ****P < 0.0001, ns P > 0.05.

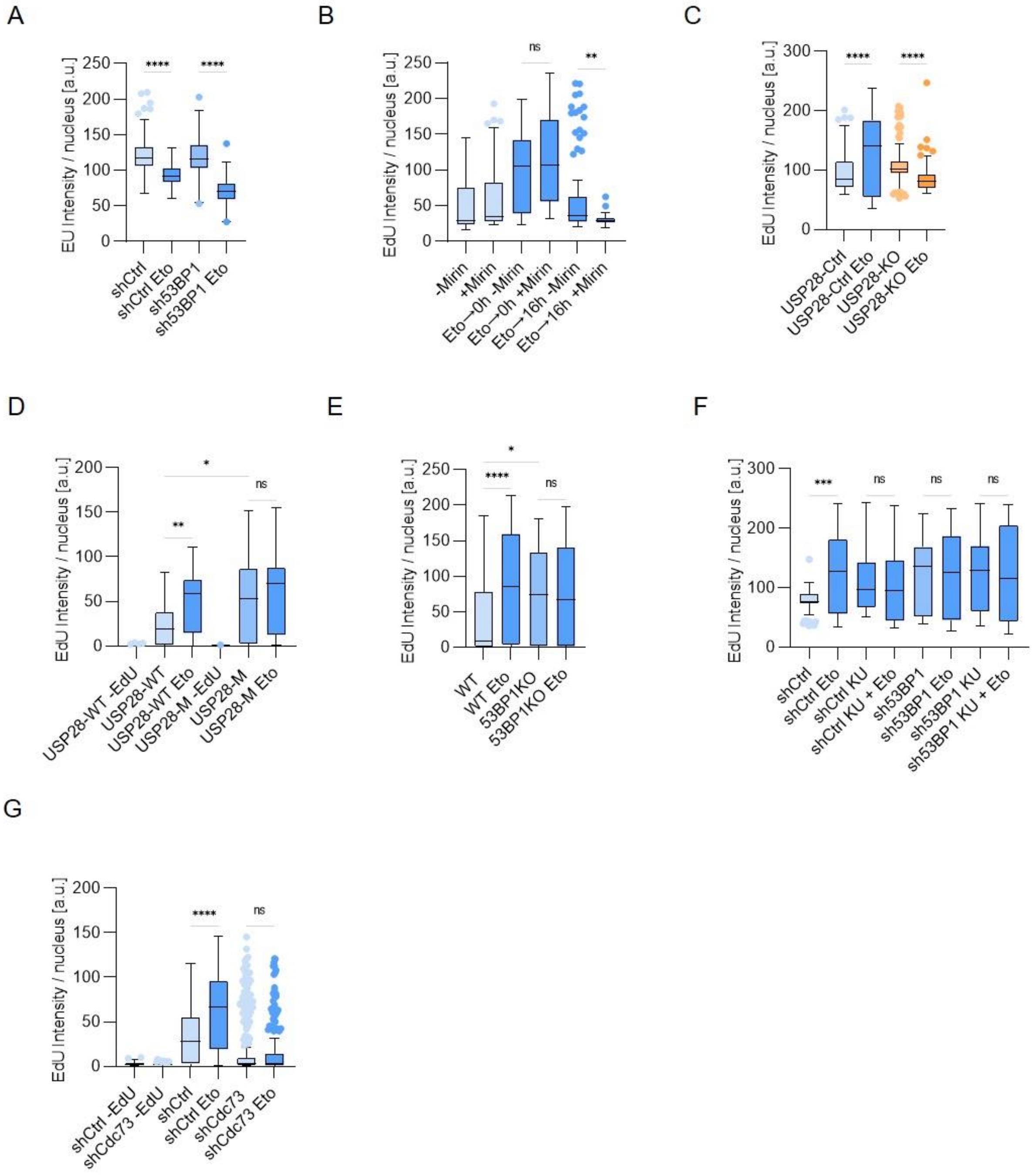

A
EU Intensity / nucleus [a.u.]
0
50
100
150
200
250
****
****
shCtrl
shCtrl Eto
sh53BP1
sh53BP1 Eto
B
EdU Intensity / nucleus [a.u.]
0
50
100
150
200
250
ns
**
-Mirin
+Mirin
Eto→0h -Mirin
Eto→0h +Mirin
Eto→16h -Mirin
Eto→16h +Mirin
C
EdU Intensity / nucleus [a.u.]
0
100
200
300
****
****
USP28-Ctrl
USP28-Ctrl Eto
USP28-KO
USP28-KO Eto
D
EdU Intensity / nucleus [a.u.]
0
50
100
150
200
*
**
ns
USP28-WT -EdU
USP28-WT
USP28-WT Eto
USP28-M -EdU
USP28-M
USP28-M Eto
E
EdU Intensity / nucleus [a.u.]
0
50
100
150
200
250
*
****
ns
WT
WT Eto
53BP1KO
53BP1KO Eto
F
EdU Intensity / nucleus [a.u.]
0
100
200
300
***
ns
ns
ns
shCtrl
shCtrl Eto
shCtrl KU
shCtrl KU + Eto
sh53BP1
sh53BP1 Eto
sh53BP1 KU
sh53BP1 KU + Eto
G
EdU Intensity / nucleus [a.u.]
0
50
100
150
200
****
ns
shCtrl -EdU
shCdc73 -EdU
shCtrl
shCtrl Eto
shCdc73
shCdc73 Eto

Fig. S7, related to Fig. 7.

(A) EU incorporation assays in HLF shCtrl/sh53BP1 cells with or without etoposide treatment (5 μM, 30 min). At least 96 cells were quantified. The data were analyzed with Kruskal-Wallis test followed by Dunn's multiple comparison of selected pairs, ****P < 0.0001.

(B) EdU incorporation assays in etoposide-treated (5 μM, 30 min followed by 0 or 16 hr release) HLF cells with or without Mirin treatment (25 mM, 2 hr for unreleased cells and 18 hr for released cells). At least 40 cells were quantified. The data for shCtrl cells were the same as the shCtrl cells in Fig. S6D. The data were analyzed with Kruskal-Wallis test followed by Dunn's multiple comparison of selected pairs, **P < 0.01, ns P > 0.05.

(C) EdU incorporation assays in HLF USP28-Ctrl/KO cells with or without etoposide treatment (5 μM, 30 min). Quantification shows data points for one representative experiment (n=2). At least 134 cells were quantified. The data were analyzed with Kruskal-Wallis test followed by Dunn's multiple comparison of selected pairs, ****P < 0.0001.

(D) EdU incorporation assays in HLF USP28-WT or USP28-M cells with or without etoposide treatment (5 μM, 30 min). Quantification shows data points for one representative experiment (n=2). At least 43 cells were quantified. The data were analyzed with Kruskal-Wallis test followed by Dunn's multiple comparison of selected pairs, *P < 0.05, **P < 0.01, ns P > 0.05.

(E) EdU incorporation assays in HeLa WT or 53BP1KO cells with or without etoposide treatment (5 μM, 30 min). At least 59 cells were quantified. The data were analyzed with Kruskal-Wallis test followed by Dunn's multiple comparison of selected pairs, *P < 0.05, ****P < 0.0001, ns P > 0.05.

(F) EdU incorporation assays in HLF shCtrl/sh53BP1 cells with etoposide (5 μM, 30 min) and KU-55933 (2 μM, 2 hr) alone or combined treatment. At least 68 cells were quantified. The data were analyzed with Kruskal-Wallis test followed by Dunn's multiple comparison of selected pairs, ***P < 0.001, ns P > 0.05.

(G) EdU incorporation assays in HLF shCtrl/shCDC73 cells with or without etoposide treatment (5 μM, 30 min). Quantification shows data points for one representative experiment (n=2). At least 102 cells were quantified. The data were analyzed with Kruskal-Wallis test followed by Dunn's multiple comparison of selected pairs, ****P < 0.0001, ns P > 0.05.

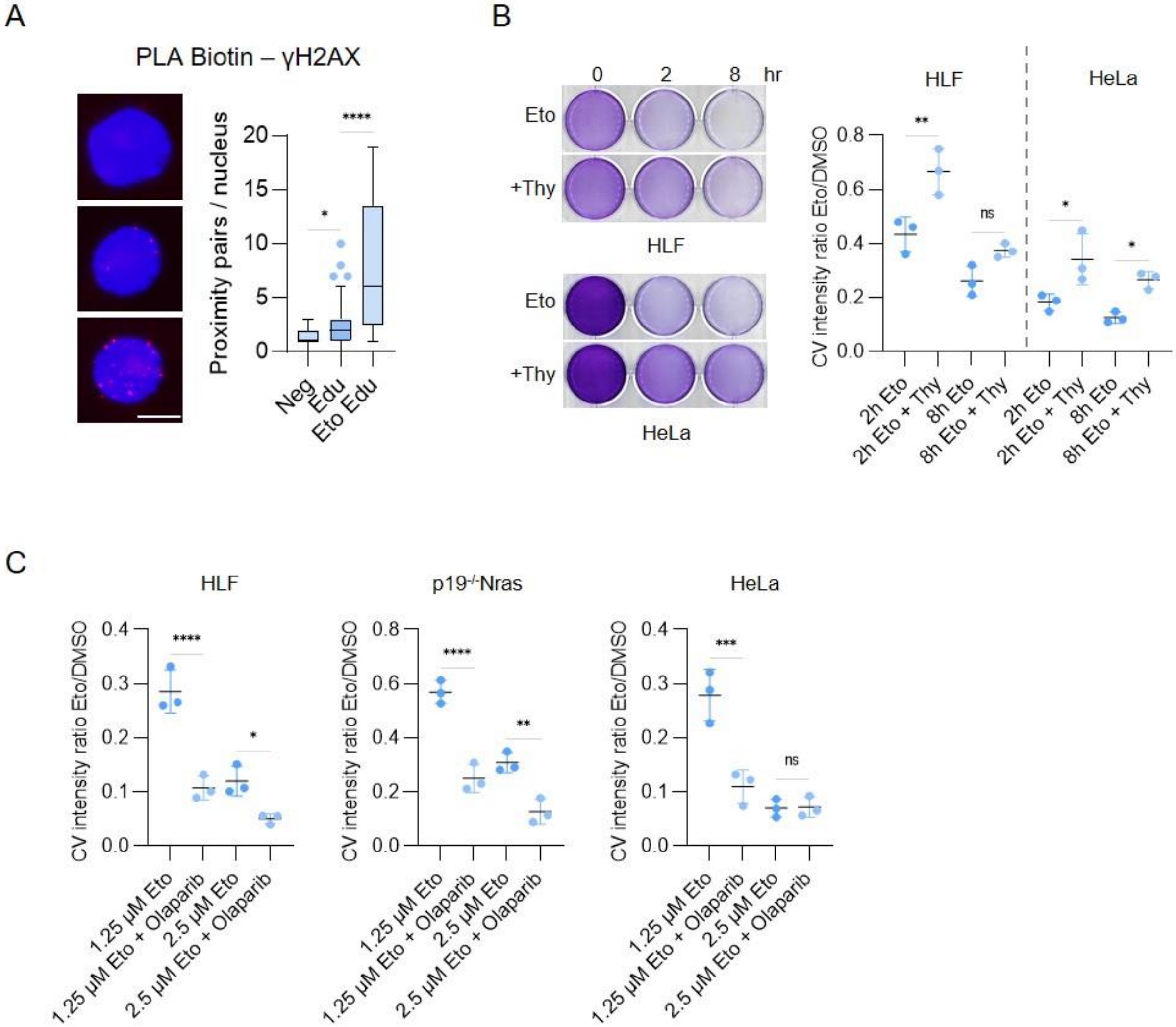

A
PLA Biotin – γH2AX
Proximity pairs / nucleus
Neg
Edu
Eto Edu
B
0
2
8
hr
Eto
+Thy
HLF
HeLa
CV intensity ratio Eto/DMSO
2h Eto
2h Eto + Thy
8h Eto
8h Eto + Thy
ns
C
HLF
p19-/-Nras
HeLa
1.25 µM Eto
1.25 µM Eto + Olaparib
2.5 µM Eto
2.5 µM Eto + Olaparib

Fig. S8, related to Fig. 8.

(A) PLA assays with antibodies against Biotin and γH2AX in HLF cells with or without etoposide treatment (5 μM, 2 hr). Quantification shows data points for one representative experiment (n=3). At least 33 cells were quantified. The data were analyzed with Kruskal-Wallis test followed by Dunn’s multiple comparison of selected pairs, *P < 0.05, ****P < 0.0001. Scale bar = 10 μm.

(B) Crystal violet staining showing etoposide (5 μM) treated HLF and HeLa cells with indicated time points with or without thymidine (2 mM, 1 hr prior etoposide treatment). Right panels show the mean of three independent biological replicates (n=3). The data were analyzed with ordinary one-way ANOVA followed by Šídák's multiple comparison of selected pairs, *P < 0.05, **P < 0.01, ns P > 0.05. Error bars denote S.D.

(C) Crystal violet staining quantifications of etoposide (1.25 or 2.5 μM, 30 min) treated HLF, $p19^{-/-}$Nras and HeLa cells with or without PARP inhibitor Olaparib (10 μM, 1 hr prior etoposide treatment) showing the mean of three independent biological replicates (n=3). The data were analyzed with ordinary one-way ANOVA followed by Šídák's multiple comparison of selected pairs, *P < 0.05, **P < 0.01, ***P < 0.001, ****P < 0.0001, ns P > 0.05. Error bars denote S.D.

Supplementary Table 1. Oligonucleotides used in the study:

Name	Sequence (5'-3')	Application
USP28-M forward	GTTAAGACCTGTGAACAGAGATGGAGGAG	Mutagenesis
USP28-M reverse	CTCCTCCATCTCTGTTCACAGGTCTTAAC	Mutagenesis
USP28-R406Q forward	CAGGAGCAAGGAGCTTATTCAAAATAAGAGAGAGTGTATTCG	Mutagenesis
USP28-R406Q reverse	CGAATACACTCTCTCTTATTTTGAATAAGCTCCTTGCTCCTG	Mutagenesis
USP28-R428T forward	CTGCAGCAAAAATTGGAAACGTATGTGAAATATGGCTCAG	Mutagenesis
USP28-R428T reverse	CTGAGCCATATTTCACATACGTTTCCAATTTTTGCTGCAG	Mutagenesis
USP28-R510S forward	CTCCTGAAGATTCTTTATCCAAGTCTAAACCACTG	Mutagenesis
USP28-R510S reverse	CAGTGGTTTAGACTTGGATAAAGAATCTTCAGGAG	Mutagenesis
USP28-R519W forward	CTAAACCACTGACATCTTCTTGGTCTTCCATGGAAATGCC	Mutagenesis
USP28-R519W reverse	GGCATTTCCATGGAAGACCAAGAAGATGTCAGTGGTTTAG	Mutagenesis
sfGFP forward	TGAGTCGGCCGGTGGATCCAATGAGCAAGGGCGAGGAG	cloning
sfGFP reverse	CCGCAGTCATCTTGTACAGCTCGTCCATG	cloning
USP28 forward	GCTGTACAAGATGACTGCGGAGCTGCAG	cloning
USP28 reverse	GAGGGGCGGATCCGTCGACATTATTTCACTGTCACAGTTGAAACTCC	cloning
sfGFP-USP28 forward	CGGCATGGACGAGCTGTACAAGGGAGGCTCTACTGCGGAGCTGCAGCAGGACG	cloning
sfGFP-USP28 reverse	CGTCCTGCTGCAGCTCCGCAGTAGAGCCTCCCTTGTACAGCTCGTCCATGCCG	cloning
h53BP1-1 forward	CCGGCCCTTGTTCAGGACAGTCTTTCTCGAGAAAGACTGTCCTGAACAAGGGTTTTTG	shRNA
h53BP1-1 reverse	AATTCAAAAACCCTTGTTCAGGACAGTCTTTCTCGAGAAAGACTGTCCTGAACAAGGG	shRNA
h53BP1-2 forward	CCGGGATACTTGGTCTTACTGGTTTCTCGAGAAACCAGTAAGACCAAGTATCTTTTTG	shRNA
h53BP1-2 reverse	AATTCAAAAAGATACTTGGTCTTACTGGTTTCTCGAGAAACCAGTAAGACCAAGTATC	shRNA
m53BP1-1 forward	CCGGCAAGTCCTTCACCCGCATTATCTCGAGATAATGCGGGTGAAGGACTTGTTTTTG	shRNA
m53BP1-1 reverse	AATTCAAAAACAAGTCCTTCACCCGCATTATCTCGAGATAATGCGGGTGAAGGACTTG	shRNA
m53BP1-2 forward	CCGGTGAATGGACAGTGACTATAAACTCGAGTTTATAGTCACTGTCCATTCATTTTTG	shRNA

m53BP1-2 reverse	AATTCAAAAATGAATGGACAGTGACTATAAACTC GAGTTTATAGTCACTGTCCATTCA	shRNA
hUSP28 forward	CACCGGAGTTGATGGTTGGCCAGTT	sgRNA
hUSP28 reverse	AAACAACTGGCCAACCATCAACTCC	sgRNA
h53BP1 forward	CACCGAACGAGGAGACGGTAATAGT	sgRNA
h53BP1 reverse	AAACACTATTACCGTCTCCTCGTTC	sgRNA
hMYC forward	TCCTACGTTGCGGTCACA	qPCR
hMYC reverse	GCTCGGTCACCATCTCCA	qPCR
hBeta-actin forward	CCAACCGCGAGAAGATGA	qPCR
hBeta-actin reverse	TCCATCACGATGCCAGTG	qPCR
hCALM2 forward	CGGACTAATTCGCCTCCTCC	qPCR
hCALM2 reverse	GTGAAGAAAGGGGTCCCGAG	qPCR
hNPM1 forward	CTCGCGAGATCTTCAGGGTC	qPCR
hNPM1 reverse	AGAACGCTGCTCCAGAGAAC	qPCR
hRPS13 forward	GAAGTGACCTCACACGTCCC	qPCR
hRPS13 reverse	CTCTTGCGACGCTGAAATGC	qPCR
hSRSF7 forward	GCGTCATCTCGTTGTTCTGC	qPCR
hSRSF7 r reverse	CATGACCCGCGTGTTAGTCT	qPCR
hMTIF2 forward	CGCTGGAAAAGGTTCTTTCCG	qPCR
hMTIF2 reverse	AGGTTGAACCAGCGCCTC	qPCR

Supplementary Table 2. Antibodies used in the study:

Name (Clone Number)	Application	Catalog Number	Lot Number	Brand
53BP1 (BP13), Mouse	IP,	MAB3802	3213923	Millipore
53BP1, Rabbit	IB, PLA	NB100-304	A5	Novus
Anti-BrdU (B44), Mouse	DNA Fiber	BD347580	8309543	Biosciences
Anti-BrdU (BU1/75 (ICR1), Rat	DNA Fiber	ab6326	GR32692464-1	Abcom
Anti-Mouse IgG-HRP	IB	7076S	38	CST
Anti-Mouse-Alexa 488	IF	4408S	21	CST
Anti-Mouse-Alexa 555	DNA Fiber	4409S	19	CST
Anti-Rabbit IgG-HRP	IB	7074S	31	CST
Anti-Rabbit-Alexa 555	IF	4413S	18	CST
Anti-Rat-Alexa 488	DNA Fiber	4416S	13	CST
Beta-Actin (AC15), Mouse	IB	A5441	-	Sigma
Biotin (33), Mouse	PLA	sc-101339	H0321	Santa Cruz
CDC34A (H-81), Rabbit	IB	sv-5616	-	Santa Cruz
CDC34B (E-6), Mouse	IB	sc-376097	H0717	Santa Cruz
CDC73 (2H1), Mouse	PLA	sc-33638	K0118	Santa Cruz
CTR9 (D1Z4F), Rabbit	PLA	12619S	1	CST
Cyclin A (B-8), Mouse	IF	sc-271682	D1818	Santa Cruz
FLAG (M2), Mouse	IB, IP	F1804-200UG	SLBN5629V	Sigma
FLAG (D6W5B), Rabbit	IB, PLA	14793S	7	CST
GAPDH (D16H11), Rabbit	IB	5174S	-	CST
GFP (B-2), Mouse	IB, PLA	sc-9996	C0217	Santa Cruz
GFP-Trap Agarose	IP	gta-20	91009001A-04	Chromotek
Histone H2AX, Rabbit	IB	2595S	8	CST
Histone H3 (96C10), Mouse	IB	3638S	8	CST
HA Tag (6E2), Mouse	PLA	2367S	5	CST
HA Tag (C29F4), Rabbit	IB, PLA	3724S	10	CST
IgG (DA1E), Rabbit	IP, PLA	3900S	45	CST
IgG (G3A1), Mouse	IP, PLA	5415S	10	CST
Jun (60A8), Rabbit	IB	9165S	9	CST
LEO1, Rabbit	C & R	A300-175A	A300-175A-3	Bethyl
MYC (C-33), Mouse	PLA	sc-42	C2713	Santa Cruz
MYC, Rabbit	IB	9402S	11	CST
MYC (D3N8F), Rabbit	IB	13987S	5	CST
MYC (Y69), Rabbit	PLA	ab32072	GR3232703-35	Abcom

PAF1, Rabbit	PLA	15441-1-AP	-	Proteintech
PCNA (PC10), Mouse	PLA	sc-56	A2422	Santa Cruz
γH2AX (Ser139), Mouse	IB, IF	sc-517348	J2120	Santa Cruz
γH2AX (Ser139), Rabbit	PLA	9718S	21	CST
pS5-RNAPII (D9N5I), Rabbit	PLA	13523S	1	CST
RNAPII (D8L4Y), Rabbit	IB	14958S	4	CST
Ubiquitin (P4D1), Mouse	IB	sc-8017	D1713	Santa Cruz
USP25, Rabbit	IB	12199-1-AP	-	Proteintech
USP28, Rabbit	IB	NBP1-82904	A41000	Novus
USP28, Rabbit	IB, IF, PLA	17707-1-AP	-	Proteintech
USP28, Rabbit	IB	HPA006778-100	2733	Sigma
Vinculin (HVIN-1), Mouse	IB	V9131-.2ML	-	Sigma
